# Nature-Related Risk Perceptions Among Vietnamese Smallholder Tea Farmers and Implications for Environmental Management

**DOI:** 10.1007/s00267-026-02534-w

**Published:** 2026-06-26

**Authors:** Thi Hoa Vu, Brett A. Bryan, Kelly K. Miller, Lai Ming Lam, Carla L. Archibald

**Affiliations:** 1 School of Life and Environmental Sciences, Melbourne Burwood Campus, Deakin University, Burwood, VIC Australia; 2 Thai Nguyen University of Agriculture and Forestry, Thai Nguyen, Vietnam

**Keywords:** Agricultural value chain, Climate-related risk, Natural capital, Tea production, Taskforce on Nature-related Financial Disclosure, TNFD, Water scarcity

## Abstract

Smallholder farmers manage a significant share of the world’s agricultural landscapes and are highly vulnerable to nature-related risks arising from climate variability, soil degradation, water scarcity, and pest and disease pressures. Sustainably managing the balance between the environment and production in such systems requires an understanding of how land managers perceive and prioritize these risks, as perceptions often influence adaptation responses. This study examines nature-related risk perceptions among Vietnamese smallholder tea farmers across 10 physical nature-related risk domains, adopting the threat appraisal component of Protection Motivation Theory. Through face-to-face structured interviews with 312 farmers in northern Vietnam, we applied multivariate regression analysis to examine the factors related to these perceptions. Farmers prioritize climate extremes and pest and disease pressures as the most immediate risks, while soil and water degradation represent an important longer-term concern. Heavy fertilizer use is associated with greater concern about soil degradation, whereas adopting irrigation is associated with lower perceived vulnerability to climate-related risks. Although subjective risk perceptions may differ from objectively assessed risks, they often play a critical role in shaping farmers’ adaptation behavior. These findings highlight the importance of incorporating smallholder risk perceptions into environmental management and broader nature-related risk assessments. Engaging smallholders directly in these processes could better capture local risks and inform nature-related risk management in agricultural systems that depend on smallholders.

## Introduction

Global agriculture relies heavily on nature, yet directly contributes to environmental pressures such as biodiversity loss, pesticide pollution, and greenhouse gas emissions (Benton et al., 2021; Tang et al., 2021). These interactions have been termed *nature-related risks*, defined as risks arising from an organization’s dependencies and impacts on nature that may affect its performance and resilience (TNFD, 2023a). Established in 2021, the Taskforce on Nature-related Financial Disclosures (TNFD) is a market-led, science-based initiative providing a framework for large and/or public organizations to identify, assess, manage, and disclose nature-related risks and opportunities (TNFD, 2023b). These risks can be classified as physical risks (e.g., extreme weather events and ecosystem degradation), transition risks (e.g., policy, market, technological, and reputational changes), and systemic risks (e.g., ecosystem collapse) (TNFD, 2023b). TNFD encourages agribusinesses to assess and disclose nature-related risks across their value chains, yet many agricultural supply chains, such as tea, depend on smallholder farmers whose context-specific risks and perceptions are poorly captured by existing risk assessment approaches, especially in developing countries (Marais et al., 2019). These frameworks often rely on coarse-resolution spatial approaches that fail to capture how farmers themselves perceive and prioritize nature-related risks, which can be shaped by local experience, farming practices, and socioeconomic conditions (Davé et al., 2025). Given the critical role of farmers’ perceptions in shaping adaptation decisions (Lamichhane et al., 2022; Petersen-Rockney, 2022), understanding smallholder farmers’ perceived nature-related risks and the factors associated with them is essential for effectively designing targeted adaptation strategies and sustaining agricultural value chains.

Smallholder farms (less than 2 ha) account for around two-thirds of all farms globally and play a key role in major perennial crops such as tea (Martinez-Nuñez et al., 2025; Ricciardi et al., 2018). They contribute around 60% of global tea production (Bolton, 2022), yet these farmers are highly vulnerable to nature-related risks. Climate change exacerbates farmers’ vulnerability by reducing their capacity to diversify livelihoods, as rising temperatures reduce crop suitability (Choquette-Levy et al., 2021; Touch et al., 2024). Vulnerability to nature-related risks is particularly high in perennial systems such as tea, where long production cycles limit farmers’ flexibility to respond to risks (Martinez-Nuñez et al., 2025). These vulnerabilities can disrupt value chains by affecting supply stability, production reliability, and product quality for downstream actors (Archer & Elliott, 2021; Wilhelm et al., 2016).

Industry sectors are increasingly expected to assess and disclose nature-related risks across value chains (Smith et al., 2024; TNFD, 2024). TNFD provides the ‘Locate, Evaluate, Assess, and Prepare’ framework (henceforth, LEAP framework), supported by tools such as ENCORE, WWF Risk Filter, and EXIOBASE to identify and assess nature-related risks and opportunities. (ENCORE, n.d.-a; TNFD, 2024; WWF, n.d.). Most tools used to quantify risk rely on spatial data at coarse resolutions, limiting their applicability to smallholder systems that are highly heterogeneous and context-specific (ENCORE, n.d.-b; Marais et al., 2019). While some frameworks assess farm-level risks using biophysical data, these are typically applied in developed countries (Ascui & Cojoianu, 2019; Smith et al., 2023). These approaches are often expert-driven and resource-intensive, limiting their accessibility to smallholders. Existing frameworks also fail to capture how farmers perceive and interpret nature-related risks, potentially limiting adaptive decision-making (Lamichhane et al., 2022; Li et al., 2024).

Despite growing interest, major gaps remain in understanding how smallholders perceive nature-related risks and the factors influencing those perceptions. Most studies focus on individual risks, such as climate or water-related risks, limiting understanding of how farmers interpret multiple and interconnected risks (Komarek et al., 2020). Recent studies examine farmers’ perception of ecosystem service changes, highlighting the role of local knowledge in recognizing environmental change (Cheng et al., 2023; You et al., 2024). However, these studies do not examine how these changes are perceived as risks to agricultural production or how socioeconomic factors influence these perceived risks (Duong et al., 2019a). Although risk perception is widely recognized as a driver of adaptation actions, less is known about how existing practices relate to perceptions about future risk (Wheeler et al., 2021). Thus, integrated research into the factors associated with smallholders’ perceived nature-related risks is essential.

Through face-to-face interviews, this study aims to understand how smallholder farmers perceive nature-related risks through their observations of ecosystem change over time, risk experiences, and perceptions of future nature-related risks. We focus on tea farms in Thai Nguyen province, Vietnam, and examine how farmers perceive nature-related risks through the threat appraisal component of Protection Motivation Theory. We explore how socio-economic characteristics, farming practices, past risk experiences, and social capital link to risk perceptions. By adopting the TNFD nature-related risk conceptual lens at the farm level through social surveys, we generate context-specific insights to support more inclusive and grounded nature-related risk assessments, which enable us to relate farmer experiences with corporate-level sustainability frameworks. We discuss the importance of managing risks equitably across agribusiness value chains via the meaningful inclusion of smallholder farmers. Understanding how farmers perceive risks and the factors associated with those perceptions is essential for developing effective, locally tailored risk identification, adaptation, and mitigation strategies (Martinez-Nuñez et al., 2025; Wilhelm et al., 2016).

## Method

### Conceptual framework

**Fig. 1:**
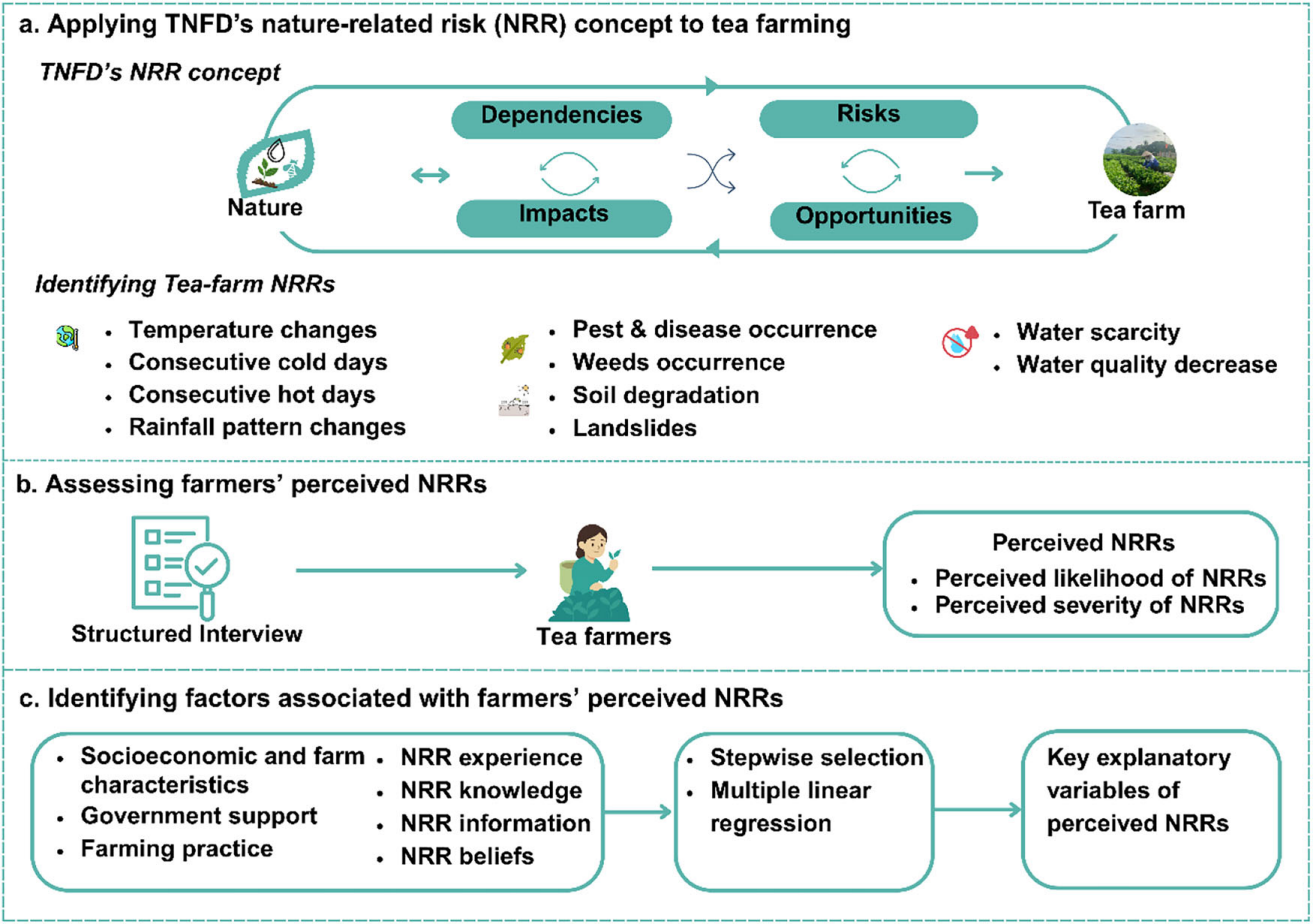
Conceptual workflow of the study. This figure illustrates: **a** the application of the TNFD’s nature-related risk (NRR) concept to tea farming to identify key NRRs; **b** the assessment of farmers’ perceived risks; and **c** the analysis of factors influencing risk perception

Nature-related risks emerge from the reciprocal relationship between an organization’s dependencies on nature and its impacts on it, degradation of the physical aspects of nature that organizations rely on can ultimately undermine their own performance and resilience (TNFD, 2023a). This study focuses on these physical nature-related risks (TNFD, 2023a), defined as risks arising from environmental degradation and the resulting loss of ecosystem services. Hereafter, nature-related risks refer specifically to physical risks. Figure 1 illustrates the study’s conceptual workflow, showing how nature-related risks were identified, assessed, and analyzed. First, the TNFD framework and relevant literature were used to identify key physical risks for tea farming. Second, drawing on the threat appraisal component of Protection Motivation Theory (PMT), farmers’ perceived risks were assessed based on their evaluations of likelihood and severity. Third, multiple linear regression was used to identify key variables associated with perceived risks.

#### Identification of nature-related risks

We identified physical nature-related risks in tea farming through a three-step process informed by selected elements of the TNFD LEAP framework, tea-production literature, and field understanding of the study area. Rather than applying LEAP as a full TNFD assessment, we used its logic to structure the identification of farm-level dependencies, impacts, and associated physical risks that may impact the smallholder farmer. The Locate element identified relevant ecosystem interfaces; Evaluate identified environmental assets, ecosystem services, and dependency–impact pathways; and Assess translated these pathways into physical risks for empirical analysis. The Prepare element was not applied, as it concerns disclosure rather than risk identification (further details in Supplementary Table S1).

**Fig. 2:**
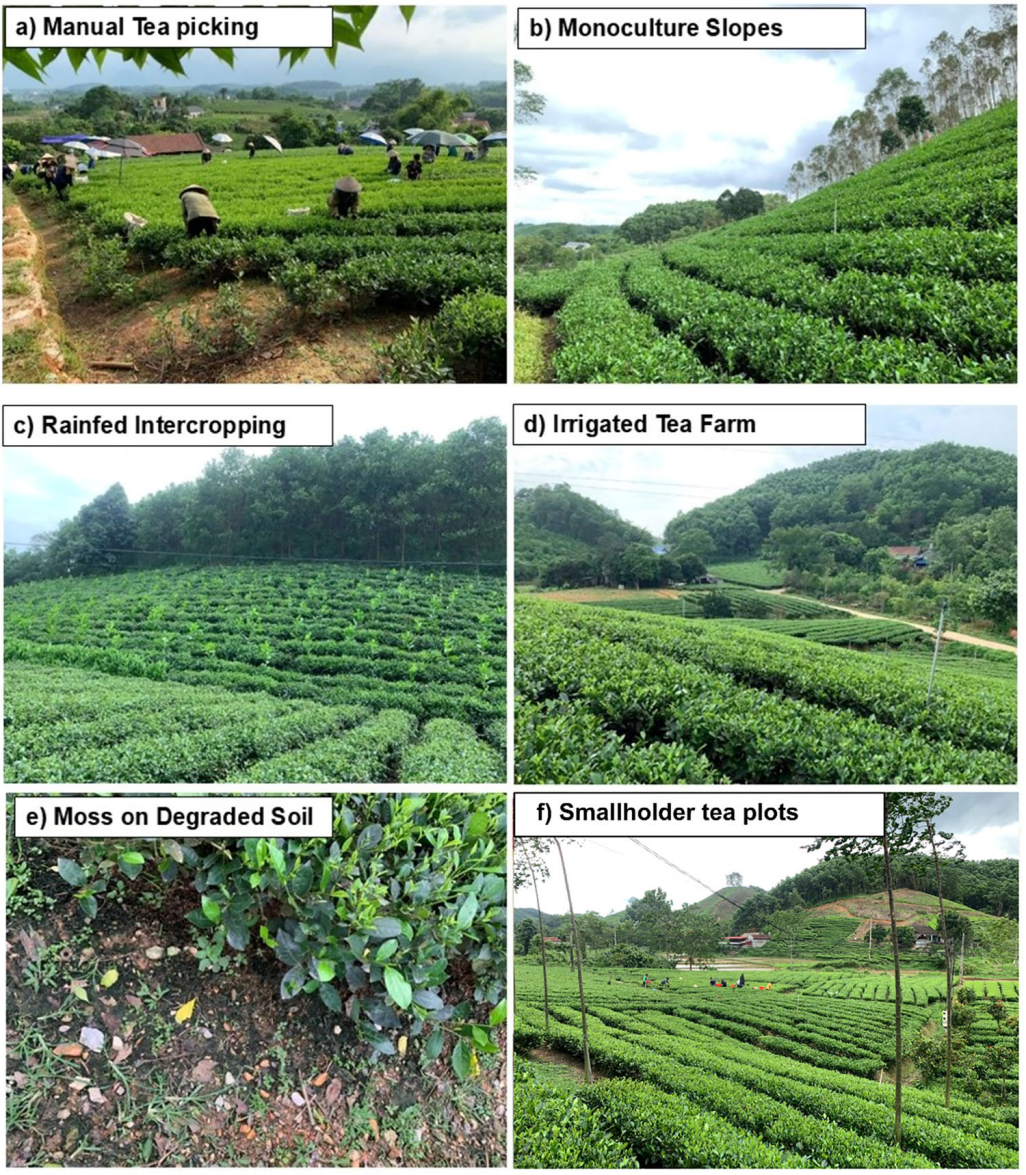
Tea farming in the study area. **a** Labor-intensive manual tea picking; **b** Sloped monoculture tea plantation; **c** Intercropped tea farm; **d** Tea farm with installed irrigation system; **e** Moss observed at the base of tea plants; **f** Smallholder tea plots

Tea production is typically located within intensive agricultural and freshwater systems. It depends on ecosystem services, including water supply, soil fertility, climate regulation, and biological control, making it vulnerable to shifting seasons, water stress, and changes in temperature and soil conditions that affect yield and quality (Jayasinghe & Kumar, 2021). For instance, tea yields decline when temperatures exceed the optimal threshold (Duncan et al., 2016). Unsustainable practices, such as overuse of chemicals and monoculture cultivation, degrade ecosystems on which tea farming depends (Le et al., 2021). Tea is often cultivated on sloping land and in monoculture systems, as illustrated in Fig. 2, which shows key characteristics of tea farming systems in the study area. Similar perennial cropping systems on steep slopes in northern Vietnam have been reported to experience soil losses ranging from 39.55 to 63.37 t·ha⁻¹·year⁻¹ (Nguyen & Pham, 2018).

This dependency–impact pathway led to nature-related risks affecting tea production: water scarcity, water quality decrease, soil degradation, landslides, increased weed occurrence, increased pest and disease occurrence, temperature changes, rainfall pattern changes, increased consecutive cold days, and increased consecutive hot days. Our results are organized into four thematic risk categories: water-related, soil-related, pest–disease–weed–related, and climate-related risks.

#### Perceived Nature-related Risk Measurement

The TNFD LEAP framework supports nature-related risk assessment by identifying dependency and impact pathways through scientific metrics, spatial data, and objective risk indicators. These are often constructed using coarse national or global-scale datasets and tend to generalize local risk processes. The threat appraisal component of Protection Motivation Theory complements this by capturing how farmers evaluate the likelihood and severity of those risks in their own local farming context. Farmers’ perceptions may complement externally derived assessments by revealing place-based experience, differ from objective indices in how risks are prioritized, or challenge expert-based assessments when externally identified risks are not perceived as immediate or severe (Eitzinger et al., 2018; Wang et al., 2025). Divergence between expert-based and farmer-based assessments can weaken interventions designed without farmer input (Eitzinger et al., 2018). Integrating farmers’ perceptions into TNFD-informed assessments can therefore make nature-related risk assessment more locally grounded and relevant to smallholder agricultural systems. In this study, we apply PMT threat appraisal to measure farmers’ perceived nature-related risks in terms of perceived likelihood and severity.

Protection Motivation Theory, originally developed by Rogers (1975), explains how fear appeals, defined as information about a threat and ways to reduce its impact, motivate health-protective behaviors. It has since been revised and widely applied to environmental and climate-related risks (Kothe et al., 2019; Milne et al., 2000). The theory conceptualizes behavioral responses to threats through threat appraisal and coping appraisal. Threat appraisal refers to how individuals evaluate a risk, including its perceived severity, perceived vulnerability, and fear, whereas coping appraisal concerns the evaluation of potential responses, including their effectiveness, feasibility, and associated costs (Milne et al., 2000).

We focus on the threat appraisal process to measure farmers’ perceived nature-related risks through perceived risk likelihood and severity. Perceived risk likelihood captures farmers’ perceived vulnerability. Perceived severity captures farmers’ evaluation of the potential impacts on tea production, with fear assumed to be reflected within this component (Dang et al., 2014; Duong et al., 2019b). The framework is adapted to longer-term and complex risks by disaggregating them into specific risk types relevant to tea production (Identification of nature-related risks), rather than treating them as a single, abstract concept. Some of these risks have already been experienced by farmers, thereby reducing psychological distance and enabling more grounded evaluations of future risks.

### Case Study Area

**Fig. 3:**
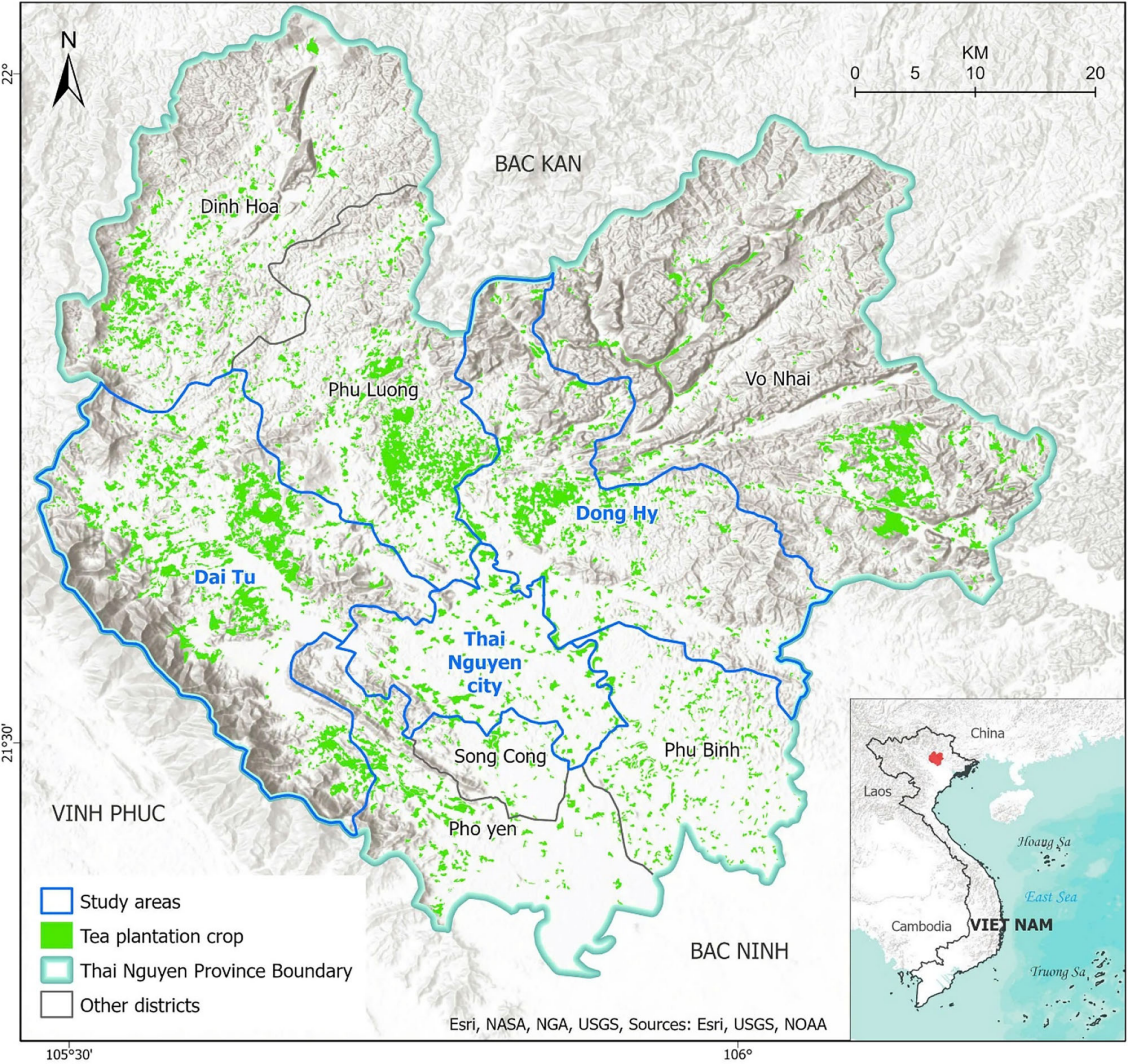
Map of the study area in Thai Nguyen province, northern Vietnam, with tea crop extent. The map reflects administrative boundaries in place at the time of data collection (2024). Tea crop data from the Thai Nguyen Department of Natural Resources and Environment (2021)

The value of Vietnam’s annual tea production is around USD 200 million annually, underscoring the crop’s significance to the national economy (Van Ho et al., 2019). Approximately 130,000 hectares are under cultivation for tea, primarily in the northern mountainous regions and the Central Highlands, and the tea sector supports the livelihoods of around 400,000 smallholders (Le et al., 2021). This study focuses on Thai Nguyen province (population 1.34 million; 3563 km² (Quang Doan et al., 2024), a mountainous region in northern Vietnam cultivating 22,027 hectares of tea and producing 230,903 tonnes of fresh leaves annually (Le et al., 2021). We focused on three key tea-producing districts: Dai Tu, Dong Hy, and Thai Nguyen City, as shown in Fig. 3. Administrative units reported in this study reflect the boundaries in place at the time of data collection in 2024 (Fig. 3).

### Data Collection

We conducted face-to-face interviews with farmers using a structured questionnaire, comprising five main sections: farm and household characteristics; past risk experiences; perceived risks; adaptation strategies; and other influencing factors, including information sources, beliefs, and institutional support. Most questions used a 5-point Likert scale, with open-ended items to capture qualitative insights. The questionnaire was developed through a comprehensive literature review and pre-tested with 15 farmers to ensure clarity and contextual suitability. Human ethics approval was obtained from the Deakin University Human Research Ethics Committee (Human Ethics Approval #2024-204).

Farmers were randomly selected from a list of tea farmers provided by local authorities from Dai Tu district, Dong Hy district, and Thai Nguyen city in Thai Nguyen province, Vietnam. This list was compiled by local authorities from records of tea-farming households in the study area and is commonly used as a sampling strategy for agricultural research in Vietnam (Bui & Nguyen, 2021; Quang Doan et al., 2024). We arranged appointments by phone, contacting 350 farmers, of whom 322 agreed to participate. Four trained researchers from Thai Nguyen University of Agriculture and Forestry conducted the interviews (each ~one hour) during June-July 2024 at community halls or farmers’ homes. Before starting, we provided each participant with a plain-language statement and a consent form. In total, 322 interviews were completed, and 312 were used for analysis after excluding 10 incomplete interviews during data cleaning.

### Variables and Measurement

#### Construction of Perceived Risk Variables

We operationalized perceived nature-related risks using the threat appraisal component of Protection Motivation Theory, in which perceived risk reflects farmers’ assessments of the likelihood and severity of potential impacts. Accordingly, we calculated perceived risk as the product of perceived likelihood and perceived severity, and this was done for each of the ten nature-related risks (Eq. 1). To capture overall risk perception, we calculated the overall perceived risk for each farmer as the mean across the ten risk types (Eq. 2). We used these individual-level measures (${R}_{{ij}}$ and $O{R}_{i}$) as dependent variables in separate regression models to examine factors influencing risk-specific and overall perceived risks.

*Farmer’s perceived specific nature-related risk was calculated as*:

1 $${R}_{{ij}}=\,{L}_{{ij}}\,\times \,{S}_{{ij}}$$

where ${R}_{{ij}}$ is the perceived risk score; ${L}_{{ij}}$ is perceived likelihood, and ${S}_{{ij}}$ is perceived severity for farmer ${i}$ for risk j. As perceived likelihood and severity were each measured on five-point Likert scales, the resulting risk score ranges from 1 to 25, where higher values indicate greater perceived risk arising from both higher likelihood and greater severity.

Farmer’s perceived overall nature-related risk was calculated as:

2 $${{OR}}_{i}=\,\frac{\mathop{\sum }_{j=1}^{10}{R}_{{ij}}}{10}$$

where $O{R}_{i}\,$ is the overall perceived risk for farmer i, calculated as the means of perceived risk scores across the ten nature-related risks.

Although perceived likelihood and severity were measured using five-point Likert scales, their product was treated as a continuous variable in the analysis. This approach implicitly assumes interval properties of the scales, which is common in applied survey-based research, and is consistent with studies that combine likelihood and severity to measure perceived risk (Dang et al., 2014; Duong et al., 2019b; Walpole & Wilson, 2021).

For descriptive purposes, we calculated mean perceived risk scores across farmers for each risk type and for the overall risk index. We used these measures to summarize general patterns of perceived risk and did not include them in the regression models (Table 3).

#### Explanatory Variables of Perceived Risk

We adopted a deductive approach to identify factors influencing farmers’ perceptions of nature-related risks, drawing on previous studies on climate change and agricultural risk perception. These variables were organized into six main categories: socioeconomic characteristics, farm characteristics, risk experience, current farming practices, information, belief, and institutional support (Table 1).

**Table 1:** Variables to Measure Risk Perceptions and Factors Influencing Risk Perception

Variables	Short Description	Scale
Perceived risk likelihood	Perceived likelihood of 10 nature-related risks in the next 20 years.	5-point Likert scale (No chance of risk to Very high chance)
Perceived risk severity to tea production	Impact of 10 nature-related risks on tea production over the next 20 years.	
Experienced nature-related risk impacts	Negative impacts of 10 nature-related risks on their tea production.	
Experienced changes in ecosystem services	Changes in environmental conditions over time.	3-point scale (Decreased, No Change, Increased)
Farming practices		
*Chemical Fertilizer Use*	Share of chemical fertilizer in total fertilizer use	Percentage
*Chemical Pesticide Use*	Share of chemical pesticides in total pesticide use	Percentage
*Organic/Biofertilizer Use*	Share of organic/biofertilizer in total fertilizer use	Percentage
*Organic Pesticide Use*	Share of organic pesticide in total pesticide use	Percentage
*Tea Farming Composition Type*	The structural composition of the farm is based on land use.	
*Current adaptation actions*	Reported adaptation measures.	Yes (1); No (0)
Knowledge	Knowledge of nature-related risks.	5-point Likert scale (Strongly disagree to Strongly agree)
Government support	Government support for nature-related risk adaptation practices.	
Information received	Being informed of nature-related risks.	
Information sources	Usefulness of information about nature-related risks.	
Beliefs	Beliefs in nature-related risks.	

Farmers’ past experiences with nature-related events were assessed through observed changes in ecosystem services and the perceived severity of past risk impacts. Observed changes in ecosystem services capture farmers’ perceptions of environmental conditions over time (e.g., changes in soil quality or pest occurrence). Experienced risk impacts refer to the realized negative effects of past risks on tea production. In contrast, perceived risk (the dependent variable) is forward-looking and captures farmers’ assessments of the likelihood and severity of potential impacts over the next 20 years. While these variables are related, they represent conceptually distinct dimensions, which supports a clearer interpretation of the associations observed in the analysis.

We also considered farmers’ knowledge of environmental threats, the information they received, and their belief in the relevance of those risks. We measured governmental support by asking farmers whether they received various forms of government support to manage nature-related risks. We included variables on current farming practices, such as the use of input chemicals and current adaptation strategies. These explore whether farmers’ existing practices shape their views on nature-related risks, which has received less attention in previous studies (Wheeler et al., 2021). Socio-economic and farm characteristics included age, education, household size, land area, and livelihood diversification, consistent with prior research on drivers of risk perception (Bitew & Minale, 2025; Dang et al., 2014; Duong et al., 2019b; Savari et al., 2025).

To ensure applicability to the local context, the selected variables were further refined through pre-testing and field engagement with farmers, following previous studies that emphasize the need to contextualize theoretically informed variables rather than applying them without contextual validation (Bryan et al., 2015; Lamichhane et al., 2020). A complete list of variables, measurement scales, and references for factors influencing risk perception is provided in Supplementary Table S2. Explanatory variables were drawn from the same categories and tailored to each risk (see Supplementary Table S3).

### Data Analysis

#### Quantitative Data Analysis

After identifying the set of explanatory variables for each risk (See Explanatory variables of perceived risk), we used multiple linear regression to examine their association with each risk and with overall perceived risk. The perceived risk score for each risk (${R}_{{ij}}$) (Eq. 1) and the overall perceived risk for each farmer ($O{R}_{i}$) (Eq. 2) are dependent variables in the regression models. We assessed the distribution of risk perception variables using skewness, kurtosis, and Shapiro–Wilk tests. Skewness and kurtosis indicated approximately symmetric distributions for most variables. Although the Shapiro–Wilk tests were significant, we attribute this to the sample size and the test’s sensitivity and therefore recommend interpreting these results with caution (see Supplementary Table S4). Overall, these tests support the use of linear regression models.

Given the relatively large number of explanatory variables relative to the sample size (*n* = 312), we used backward stepwise selection based on Akaike’s Information Criterion (AIC) to identify a parsimonious set of variables associated with perceived risk. Starting from the full specification, variables were sequentially removed, and the final model retained only those that improved model performance, stopping when no further reduction in AIC was achieved. This iterative approach is commonly used in studies of farmers’ behavior and decision-making (Duong et al., 2021; Wool et al., 2023). To account for multiple hypothesis testing across models, we applied false discovery rate (FDR) adjustments to the p-values, reducing the likelihood of identifying spurious associations while maintaining statistical power (Benjamini & Hochberg, 1995). Equation 3 presents the general specification of the regression model:

3 $${Y}_{i}={\beta }_{0}+{\beta }_{1}{X}_{i1}+{\beta }_{2}{X}_{i2}+{\beta }_{3}{X}_{i3}+\ldots {\beta }_{K}{X}_{{iK}}+{\epsilon }_{i}$$

where ${Y}_{i}\,$ represents the dependent variable (i.e., ${R}_{{ij}}$ or $O{R}_{i}$); ${\beta }_{0}\,$ is the intercept; ${X}_{{ik}}$ denotes the value of the *k*-th explanatory variable for farmer *i*; ${\beta }_{k}\,$ is the corresponding coefficient; *k* = 1,2,…, K, where K is the total number of explanatory variables included in the model and ${\epsilon }_{i}\,$ is the error term.

We conducted all analyses in R version 4.4.2 using multiple linear regression implemented via the *lm* function. We performed backward stepwise selection using the stepAIC function from the MASS package, alongside supporting packages for data preparation, analysis, and visualization.

#### Qualitative Data Analysis

To complement and enrich the quantitative data collected, we provided farmers with an important opportunity to share their perspectives on the reasons underlying changes in ecosystem services and the impacts of nature-related risks on their tea production. Farmers’ responses were collected through open-ended survey questions, and illustrative quotes are presented to highlight views. We employed a deductive coding approach using Microsoft Excel. This approach was designed to complement the quantitative assessment of pre-identified nature-related risks rather than to generate new categories inductively. Deductive qualitative research enables researchers to use existing theory to interpret meaning, processes, and participant narratives (Fife & Gossner, 2024). We developed coding categories from the TNFD-informed conceptual framework, relevant literature, and the predefined risk domains included in the structured questionnaire. We then categorized responses into themes related to (i) changes in ecosystem services and (ii) the impacts of nature-related risks on tea farming. For consistency, the lead author conducted all coding using this framework.

## Results

### Smallholder Farmers’ Socio-economic Characteristics

The final sample comprised 312 smallholder tea farmers; 54% female, 46% male. The average age was 53.3 years (SD = 14.3), with a range of 24 to 81 years. Household size varied from 1 to 9 members, with an average of 4.6 (SD = 2.1). Tea farm sizes ranged from 0.04 to 4.6 hectares, with an average of 0.45 hectares (SD = 0.49). Most participants had completed secondary education (43.9%) or high school (42.6%), while 7.1% had attained tertiary education and 6.4% had only primary or no formal education. In terms of annual income, 45.8% earned less than VND 50 million (approximately USD 1955), 39.4% earned between VND 50 and 100 million (USD 1955–3900), and 14.7% earned more than VND 100 million (USD 3900). Most participants had over 30 years of experience in tea cultivation (91%).

### Farmers’ Perceptions and Experiences With Nature-related Risks

#### Perceived Changes in Ecosystem Services

**Fig. 4:**
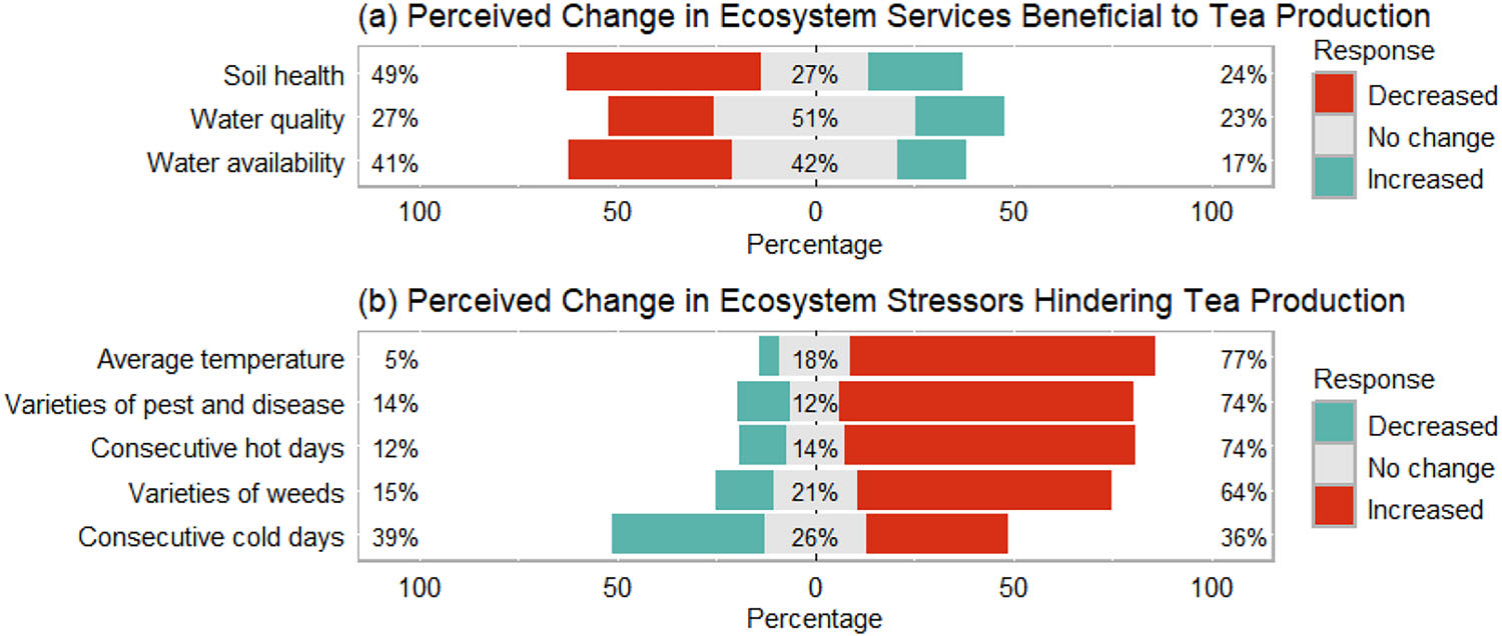
Farmers’ perceived change in ecosystem services and stressors. **a** Perceived changes in ecosystem services beneficial to tea production. **b** Perceived changes in ecosystem stressors hindering tea production. Bars show the percentage of respondents reporting that each indicator had increased, decreased, or remained unchanged

Farmers reported widespread changes in ecosystem services at the time of the interviews (June–July 2024) compared to when they began farming. Figure 4 presents farmers’ perceptions of changes in ecosystem services and environmental stressors over time. Nearly half perceived declines in soil health (49%) and water availability (41%) (Fig. 4a). Qualitative responses linked declining soil health to long-term use of chemical fertilizers, continuous cultivation, and topsoil erosion (Table 2). In contrast, 24% reported improved soil conditions, attributed to the use of organic fertilizers such as manure and bio-fertilizers. Perceptions of water quality were more mixed, with about half reporting no change and a smaller share indicating deterioration. These results indicate that declines were more commonly perceived than improvements in key ecosystem services.

**Table 2:** Summary of Themes: On Farmers’ Perceived Drivers Of Ecosystem Service Changes and Impacts of Nature-related Risks on Tea Production

Ecosystem Changes and Nature-related Risks	Drivers and Impacts of Ecosystem Change	Number of Participants (*n*)
**Farmer-perceived drivers of changes in ecosystem services**		
Perceived climate variability	Climate change	70
Increased pest and disease occurrence	Climate-driven increase in pest and disease prevalence	82
	Low-quality of fertilizer inputs and pest resistance	3
Decreased water availability	Reduced rainfall and rising temperature	21
	Increased reliance on drilled wells for irrigation	15
	Deforestation	7
	Lack of electricity for irrigation	5
Decreased water quality	Pollution from chemical fertilizers and pesticides.	10
Soil degradation	Use of chemical inputs and long-term cultivation	25
	Changes in rainfall patterns	9
Improved soil health	Use of organic/bio fertilizers	10
**Impacts of nature-related risks on tea production experienced by farmers**		
Overall impacts of nature-related risks	Yield and quality decline	77
	Increased cost/labor	22
Impacts of consecutive hot days and high temperatures	Tea wilting, leaf scorch, pest outbreaks	74
Impacts of rainfall pattern changes	Increased pest and disease occurrence: Fungal disease, root rot, and tea blister blight	69
	Soil erosion	10
	Waterlogging-induced plant loss	5
Impact of consecutive cold days	Slows tea growth	6
Impact of erratic weather	Disrupted harvest/processing, fertilizer inefficiency	6
Impact of water scarcity	Tea wilting or stunted growth	7
Impact of soil degradation	Root damage and weak plants	5
	Poor soil health increases pest/disease susceptibility	10

Farmers also reported increased exposure to climate and biological stressors (Fig. 4b). Over 75% noted rises in temperature, consecutive hot days, and increased occurrence of pests and diseases. These were often perceived as resulting from changing climate conditions that favor pest development. Weed prevalence also increased (64%). Consecutive cold days were the only factor more often perceived to have decreased (39%) rather than increased (36%). These findings suggest that climate and biological stressors are widely perceived as intensifying, contributing to growing environmental pressure on tea production systems.

#### Nature-related Risk Experiences

Tea farmers commonly reported that nature-related risks disrupted tea production by reducing yield stability, lowering quality, and increasing labor demands (Table 2). Farmers elaborated on these impacts in their own words:

Farmer A: “*Extreme heat and heavy rain severely affect tea plants, causing them to wilt or die. These conditions also lead to more pests and diseases, ultimately reducing both the quantity of tea produced and its quality, such as turning the brewed tea reddish instead of the usual green.”*

Farmer B: “*Nature-related risks significantly affect tea production by reducing yield stability, lowering quality, increasing labor costs, and making cultivation more challenging*.”

**Fig. 5:**
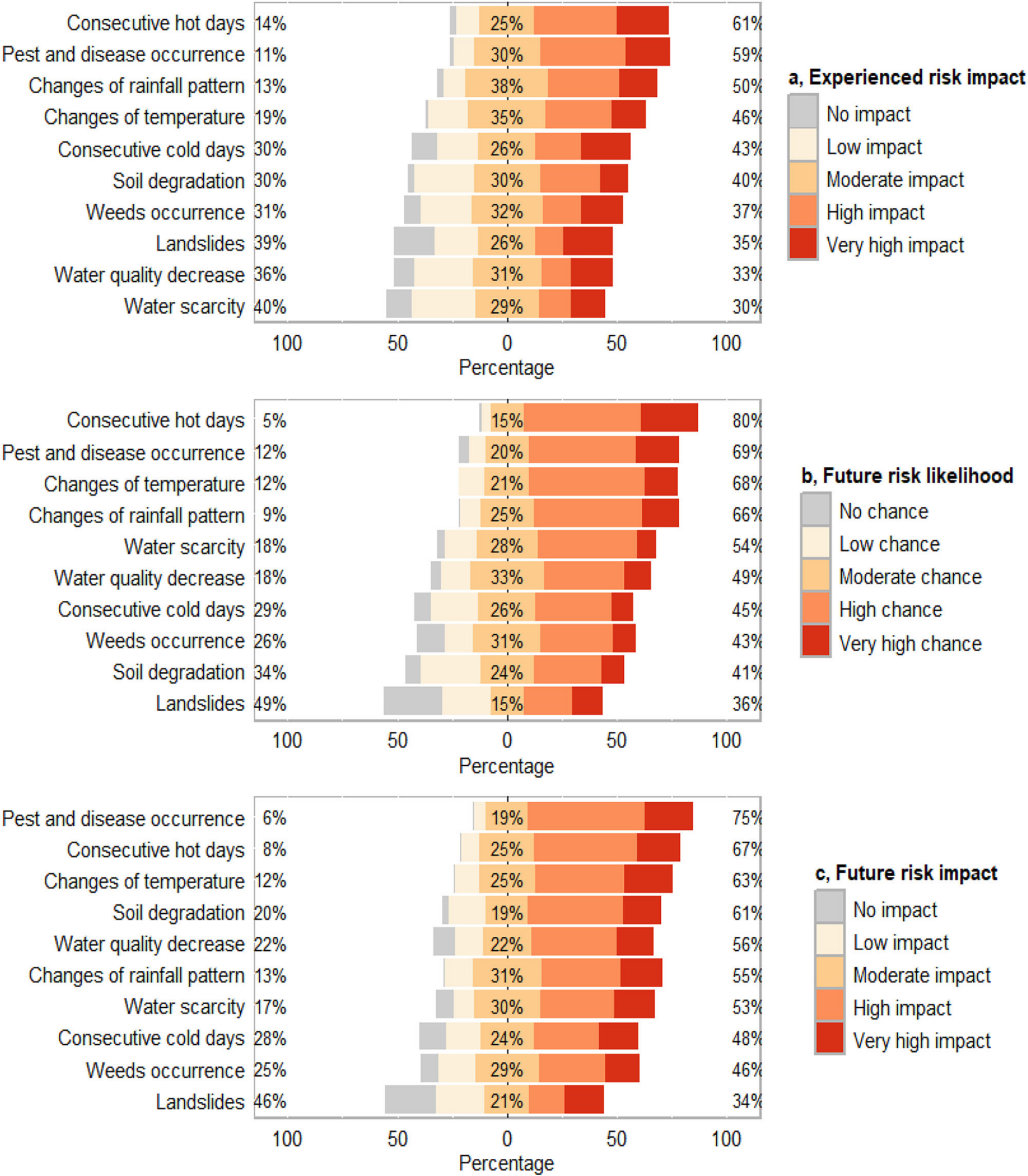
Farmers’ perception of nature-related risks. **a**, Farmers assessed the level of impact of past events on their tea production; **b**, Farmers predicted the likelihood of nature-related risk occurrence within the next 20 years; **c**, Farmers assessed the level of impact of nature-related risks if they happen within the next 20 years

These concerns were reflected in the specific farmers’ risk severity ratings in the survey, as summarized in Fig. 5, which presents past impacts, expected likelihood, and future severity of nature-related risks. Consecutive hot days were rated as the most severe (61% high or very high impact), followed by pest and disease occurrence (59%), changes in rainfall patterns (53%), temperature changes (46%), consecutive cold days (43%), landslides (41%), and soil degradation (40%). Water-related risks, including water quality decline (31%) and water scarcity (30%), were rated the lowest among the listed risks but still posed concerns for many farmers. These results indicate that farmers perceive climate extremes and biological stressors as having the most immediate and severe impacts on tea production.

Farmers explained that consecutive hot days caused leaf scorch, wilting, and pest outbreaks, while heavy rainfall led to fungal diseases, root rot, and waterlogging. Consecutive cold days were said to delay bud formation and reduce productivity. Erratic weather disrupted spraying and fertilizing, increasing input costs. A few farmers described positive effects, such as extended harvest periods due to warmer weather and shorter winters, or improved growth with adequate rainfall, though these were less commonly reported. An extended table with comments is available in the Supplementary Table S5.

#### Perceptions of Future Nature-related Risks

Most tea farmers perceived a high or very high likelihood of climate-related risks within the next 20 years (Fig. 5b). Consecutive hot days (80%) and pest and disease occurrence (69%) were most frequently identified, followed by temperature and rainfall-related risks. Water-related risks were perceived as moderate, while cold days, soil degradation, and landslides were less frequently considered highly likely, although still noted by a substantial proportion of farmers. This pattern suggests that farmers expect climate and biological risks to intensify more than other types of risks in the future (Fig. 5b).

Regarding expected impact, 75% of farmers believed pest and disease occurrence would have a high or very high impact on tea production, followed by consecutive hot days (67%) and temperature changes (63%). Water-related risks were again rated as moderate, while weeds and landslides were perceived as having comparatively lower impacts. These responses indicate that biological and climate-related risks are expected to have the most severe consequences for tea production (Fig. 5c).

Perceived risk was measured by combining farmers’ ratings of the likelihood and impact of each nature-related risk, ranging from 1 to 25, with higher scores indicating greater risk perception (Table 3). Based on these combined scores, consecutive hot days (mean = 15.21) and pest and disease occurrence (mean = 14.67) emerged as the most critical risks, followed by temperature change (13.96) and rainfall pattern change (13.65). Water scarcity (12.21) and water quality decrease (11.79) were moderate risks. Landslides received the lowest combined score (8.21), indicating a comparatively lower level of concern. These results indicate a clear prioritization of climate and biological risks over soil- and water-related risks in farmers’ perceptions.

**Table 3:** Mean and Standard Deviation of Perceived Probability, Perceived Severity, and Specific Perceived Risk for Various Nature-related Risks

Type of Risks	Perceived Probability		Perceived Severity		Specific Perceived Risk	
	Mean	SD	Mean	SD	Mean	SD
Consecutive hot days increase	4.00	0.82	3.78	0.87	15.21	5.05
Pest and disease occurrence	3.72	1.00	3.91	0.82	14.67	5.37
Changes of temperature	3.71	0.87	3.72	0.95	13.96	5.15
Changes in rainfall patterns	3.73	0.86	3.60	0.97	13.65	5.31
Water scarcity	3.42	0.96	3.46	1.14	12.21	5.80
Water quality decreases	3.38	1.01	3.40	1.19	11.79	5.91
Soil degradation	3.11	1.13	3.56	1.06	11.17	5.63
Weeds occurrence	3.15	1.17	3.28	1.17	10.71	6.12
Consecutive cold days increase	3.18	1.11	3.26	1.27	10.70	6.14
Landslides	2.75	1.42	2.83	1.42	8.21	6.61
Overall Nature-related risks					12.23	3.17

Perceived probability and perceived severity were rated on 5-point Likert scales (1 = lowest, 5 = highest). Specific perceived risk was calculated as probability × severity, ranging from 1 to 25, with higher scores indicating greater risk perception

### Factors Influencing Farmer Perceptions of Nature-related Risk

**Fig. 6:**
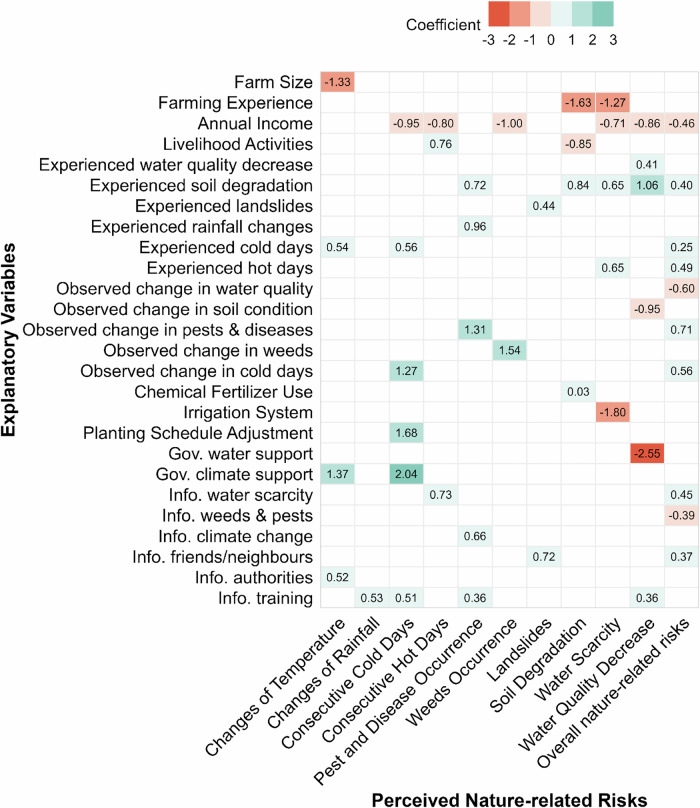
Factors associated with farmers’ perceived nature-related risks based on multiple regression models. Each cell shows the regression coefficient for an explanatory variable (rows) and a specific farmer’s perceived risk (columns), with values displayed inside the cell (rounded to two decimal places). Red shading indicates negative regression coefficients, while green shading indicates positive regression coefficients. Only explanatory variables that remained statistically significant after false discovery rate correction are shown

Using multiple regression models, we identified key factors influencing tea farmers’ perceived nature-related risks. Figure 6 presents the explanatory variables significantly associated with farmers’ perceived nature-related risks across different risk types. Prior risk experience and information-related factors were most consistently associated across risk themes, while socio-economic and management variables were important for specific risks. Model fit was modest (R² = 0.033–0.237; Table 4). These results reflect statistical associations in cross-sectional data and should not be interpreted as causal. The following paragraphs summarize variables significantly associated with each risk (p < 0.05 after FDR correction); full model results are provided in Supplementary Table S6.

**Table 4:** Coefficient of Determination (R²) for Models of Perceived Nature-related Risks

Perceived risks	R^2^
Changes in Rainfall	0.083
Changes of Temperature	0.058
Consecutive Cold Days	0.140
Consecutive Hot Days	0.054
Landslides	0.033
Overall nature-related risks	0.237
Pest and Disease Occurrence	0.152
Soil Degradation	0.112
Water Quality Decrease	0.174
Water Scarcity	0.098
Weeds Occurrence	0.103

The table reports the sample size (*n* = 312) and R² values, indicating the proportion of variance in perceived risk explained by each model

Perceived risk of temperature change was higher among farmers who experienced increases in consecutive cold days, received information from local authorities, and obtained government climate-related support, and lower among those with larger farm sizes. Rainfall patterns change risk perception was positively associated with receiving information from training courses. Perceived risk of consecutive cold days was higher among farmers who observed and experienced impacts of an increase in consecutive cold days, received government climate support, valued training course information, adjusted their planting schedules, and lower among farmers with higher income. For consecutive hot days, perceived risk was lower among those with higher incomes and higher among farmers who received information about water scarcity and engaged in more diverse livelihood activities, with most relying on agriculture.

Perceived risk of pest and disease occurrence was significantly positively associated with those who experienced soil degradation, were impacted by rainfall pattern changes, reported increased pest and disease occurrence, and received climate change information. Perceived risk of weed occurrence was significantly higher among those who observed increased weed prevalence and lower among those with higher income.

Perceived risk of soil degradation was significantly higher among farmers who had experienced soil degradation or applied chemical fertilizer, and lower among those with greater farming experience or more diverse livelihood activities. The perceived risk of landslides was significantly higher among those who experienced landslides and received information from friends, relatives, and neighbors.

Perceived water scarcity was significantly higher among farmers who had experienced an increase in consecutive hot days and soil degradation, and lower among those with higher incomes, greater farming experience, and access to irrigation systems. Perceived risk of water quality decline was higher among farmers reporting soil degradation, direct experience of water quality deterioration, and receiving training information, and lower among those with higher income, government support for water management, and those who observed improved soil conditions compared with the past.

Overall, perceived nature-related risk was significantly higher among farmers who had experienced soil degradation, observed increases in consecutive hot and cold days, more frequent cold days, and pest and disease occurrence, received information about water scarcity, and obtained information from friends, relatives, and neighbors. In contrast, overall perceived risk was significantly lower among farmers with higher incomes, those who observed better water quality, and those who received information about weeds and pests. These associations suggest that farmers who had direct experience with multiple environmental stressors or greater exposure to certain types of risk-related information were more likely to perceive higher overall nature-related risk, whereas better environmental conditions and greater socio-economic capacity were associated with lower overall concern.

## Discussion

Global supply chains for tea and other perennial crops are underpinned by smallholder farmers’ ability to manage risks in a changing environment. This study reveals how smallholder tea farmers in northern Vietnam perceive nature-related risks and the factors shaping them. Farmers identified climate and pest-related risks as the most pressing. Perceived nature-related risks were associated by both the occurrence and the severity of risks, as well as interconnections among risks. Prior risk experience, current farming practices, access to information, and socio-economic conditions were significantly associated with these perceptions. These findings suggest potential implications for incorporating smallholder farmer perspectives into upstream risk disclosure practices, designing inclusive adaptation strategies, and strengthening resilience within agri-food value chains.

### Nature-related risks in smallholder tea farming: insights from farmers’ perceptions

Farmers most frequently reported changes in climate conditions and pest and disease pressures, identifying them as the most serious future nature-related risks. Rising temperatures, more frequent hot days, and erratic rainfall were perceived as intensifying, consistent with other studies in Vietnam (Dang et al., 2014; Thuy & Anh, 2021; Tran et al., 2022). We found that hot days were considered the most serious future risk, with 80% of farmers anticipating further intensification. Farmers linked these stressors to a decline in yield and quality. This is consistent with evidence that higher temperatures reduce tea yields, particularly when temperatures exceed optimal thresholds for tea growth (Duncan et al., 2016). Higher temperatures may also reduce tea quality by lowering key compounds such as catechins and antioxidants (Jayasinghe & Kumar, 2021). Pest and disease outbreaks followed a similar pattern. Farmers reported more frequent infestations that were increasingly difficult to manage. These stressors were perceived to reduce crop health, increase labor demands, and production costs. These perceptions align with findings from tea-growing regions (Ho et al., 2018), and other agro-ecosystems (Duong et al., 2019a; Komarek et al., 2020).

In contrast, farmers perceived soil degradation, water scarcity, and water quality decline as less immediate threats compared to climate and biological risks. These risks are often classified as chronic, whereas climate extremes and pest outbreaks are more acute and visible, which may explain their higher prioritization (TNFD, 2023a). Farmers linked chronic risks to overuse of fertilizer, erosion, groundwater extraction, and chemical runoff. While many farmers reported soil degradation, some noted improvements from using organic fertilizers. Although water pollution was seen as less urgent, concern about its long-term impact is increasing, as also observed among rice farmers in the Mekong Delta (Tran et al., 2022). This emphasizes the importance of supporting farmer-led soil and water management for long-term resilience.

Applying the concept of nature-related risk, which considers how farms depend on and affect nature, alongside farmers’ farming experiences, helps identify priority risks and areas for improvement. This understanding is crucial for the development of practical and locally grounded adaptation strategies. To support such strategies, it is also necessary to understand the factors associated with farmers’ perceptions of these risks.

### Factors Influencing Nature-related Risk Perceptions

We found that farmers’ perceptions of nature-related risks were associated with past experiences, access to information, farming practices, and socio-economic conditions. Observed negative ecosystem changes and experience of impacts from nature-related risks were consistently associated with higher perceived risks across multiple risk types. Similar patterns have been reported in studies of flood risk perception, where individuals with greater exposure to past flood events report higher perceived risk of future flooding (Rufat & Botzen, 2022). Farmers experiencing soil degradation and rainfall variability also reported higher perceived risk of pest and disease occurrence, reflecting how climatic risks often intersect with challenges such as pest pressure and declining yields (Bitew & Minale, 2025). These findings underscore the importance of assessing agricultural risks through a holistic lens like the nature-related risk approach, rather than focusing on individual risks in isolation (Komarek et al., 2020).

Farmers’ perceptions of nature-related risks were also linked to their farming practices. Farmers often linked unsustainable practices, such as heavy fertilizer use, to greater concern about soil degradation, likely reflecting their experience with declining soil quality (Bui & Nguyen, 2021). Similar associations have been reported in other contexts, including among yam farmers in Africa (Madin et al., 2022); however, these relationships are primarily based on farmers’ observations, and causal mechanisms remain uncertain, particularly given the potential role of overapplication or long-term management practices (Madin et al., 2022). Different adaptive actions were associated with perceived risks in different ways. Farmers who used irrigation tended to report lower perceived risk of water scarcity. Conversely, farmers who adjusted planting schedules reported higher perceived risk of consecutive cold days over the next twenty years.

We also found that access to information was associated with farmers’ risk perceptions. Information from friends, relatives, and neighbors was positively associated with perceived landslide risk, highlighting the role of trust-based social networks in shaping farmers’ risk perceptions. Farmers who received training courses were more likely to report higher perceived risks of rainfall pattern changes, consecutive cold days, pest and disease occurrence, and water quality decline. Ho et al. (2018) found that information from extension training shaped farmers’ concerns, though it helped reduce worries about pest and disease risks in their case. These findings suggest that risk perception is influenced by the type, quality, and framing of the information they receive (Dang et al., 2014; Shrestha et al., 2022).

Risk perceptions among tea farmers were also influenced by socio-demographic characteristics and institutional support. Income was negatively associated with several types of nature-related risks, indicating that farmers with lower incomes tended to perceive higher levels of risk. This may reflect greater vulnerability due to limited adaptive capacity or greater dependency on farming as a livelihood (Dang et al., 2014). Farmers with greater farming experience reported lower perceived risks of soil degradation and water scarcity. This may partly reflect farmers’ accumulated knowledge and experience over time, as they adopt practices such as using organic fertilizer and observing improvements in soil conditions. Institutional support showed mixed effects. Government water management assistance was associated with lower perceived risk of water quality decline, while climate support programs were associated with higher awareness of consecutive cold days. This suggests that different forms of support can influence risk perception in different ways.

### Policy Implications: From Smallholder Farmers to Global Supply Chains

Because this study captures farmers’ perceived risks at a single point in time, the policy implications offer guidance for risk assessment and farmer engagement rather than direct evidence of adaptation behavior or value-chain governance. Farmers’ perspectives could be critical for managing nature-related risks across supply chains. Smallholders possess place-based knowledge and are often the first to detect nature-related risks. Engaging with them can offer firms an informal, but potentially effective, early warning system for emerging risks (International Finance Corporation, 2023). For example, although water scarcity was not rated as the most severe current risk in our study, farmers expressed growing concern about future shortages due to increasing groundwater extraction for tea production. Such anticipatory perception can support more proactive responses from companies, such as investment in water storage or improved sustainable irrigation. Nature-related risk assessments based solely on objective, spatially derived data may not align with farmers’ perceptions, which are shaped by experience and local context (Nagel et al., 2024). A study in China shows that areas with high objective risk may exhibit low perceived risk due to trust in infrastructure, while areas with lower objective risk may show greater perceived concern due to recent experiences (Wang et al., 2025). Understanding these differences can help align technical assessments with real-world decision-making and support more effective, context-specific interventions.

Despite increasing uptake of frameworks such as TNFD to assess nature-related risks, important gaps remain in their application in smallholder farming systems as it offers little practical guidance on engaging directly with smallholders. For example, in California’s almond industry, companies have used the TNFD framework to identify nature-related risks, such as water scarcity and pollinator decline (TNFD, 2025). However, solutions like efficient irrigation and habitat management rely entirely on farmers’ willingness and capacity to adopt new practices. Without insights into how farmers perceive these risks or what motivates their decisions, such interventions may be less effective.

To improve nature-related risk assessments in smallholder-dependent value chains such as tea, cacao, and coffee, it is essential to integrate farmer-centered approaches into the TNFD framework. Our findings suggest perceived risk likelihood and severity, prior experience and observed ecosystem changes, farming practices, information access, socio-economic conditions, and institutional support as potential entry points for integrating farmers’ perspectives. Practical approaches may include participatory risk mapping, social surveys, or partnerships with farmer organizations. While participatory approaches are already used by companies, they typically focus on farming practices and social conditions rather than risk assessment. For example, the Twinings Community Needs Assessment engages large numbers of farmers to collect such information, but does not explicitly assess nature-related risks (Twinings, 2023). Integrating frameworks such as TNFD into these approaches could help incorporate farmer perspectives into risk assessment and support more grounded and effective risk management strategies.

### Uncertainty and Limitations

While this study offers important insights into how smallholder tea farmers perceive nature-related risks, we acknowledge several limitations. First, the analysis focuses on physical nature-related risks and does not consider transition risks such as policy changes, market dynamics, and reputational risks. Including these other types of risks would provide a more comprehensive understanding of the challenges faced at the farm level. This study does not explicitly measure fear as a separate component, which may limit the extent to which affective dimensions are captured, as recent research highlights that perceived risk involves not only evaluations of severity and probability but also emotions and concerns related to hazards (Wilson et al., 2019).

Farmers’ perceptions were recorded at a single point in time and may change as environmental conditions or personal experiences evolve. Nonetheless, the study provides a valuable baseline for understanding current perceptions and can serve as a reference point for future longitudinal research. As the data are cross-sectional, the results reflect statistical associations and should not be interpreted as causal relationships. The explanatory power of some regression models was relatively limited, indicating that the models capture only part of the variation in farmers’ perceived risks and should not be interpreted as fully explaining risk perception. We also acknowledge that survey responses may overreport socially desirable behaviors. For example, studies of political participation consistently find that many non-voters misreport having voted (Ansolabehere & Hersh, 2017). In agricultural contexts, farmers may provide responses that do not fully reflect their actual practices, for example, underreporting the use of chemical fertilizers due to concerns about social desirability or regulatory scrutiny. Treating Likert-scale measures as continuous variables may introduce measurement limitations, although this approach is widely used in survey-based research.

Finally, although understanding farmers’ perceptions is vital, other factors such as economic constraints, market access, institutional support, and social networks are also important influences on farmers’ adaptation decisions. TNFD and other nature-related frameworks should consider these broader drivers alongside risk perception to design strategies that are not only technically sound but also socially and economically feasible.

## Conclusion

This study aimed to investigate how smallholder tea farmers in northern Vietnam perceive nature-related risks and the factors associated with their perceptions. Smallholders play a crucial role in agricultural value chains but remain highly vulnerable to nature-related risks, with implications that may extend beyond the farm level. Our findings indicate that farmers perceive climate stressors and pest outbreaks as the most serious risks, while concerns about soil and water also remained important. By examining how farmers perceive the likelihood and severity of nature-related risks, this research highlights that risk perception reflects not only physical hazards but also socio-economic conditions, farming practices, and institutional support. Insights from this research also suggest that frameworks such as the TNFD, which currently emphasize financial risks and spatial assessments, should better integrate smallholder engagement through participatory approaches to understand local risk perceptions and socio-economic contexts. Understanding both nature-related risks and how farmers interpret and respond to them is vital for designing interventions that are locally relevant and more likely to be adopted. Future research should examine additional factors, beyond risk perception, that influence smallholders’ intentions and capacity to adopt adaptation measures.

### Declaration of Generative AI and AI-assisted Technologies in the Writing Process

During the preparation of this work, the author(s) used ChatGPT Version 5 to proofread the manuscript and improve its readability and language. After using this tool/service, the author(s) reviewed and edited the content as needed and took full responsibility for the content of the published article.

## Supplementary information

Supplementary information

## Data Availability

The raw survey data are not publicly available due to ethical considerations (Deakin University Human Research Ethics Committee Human Ethics Approval #2024–204) as they may contain information that could compromise participant privacy.
