# Supplementary material for: Nature-Related Risk Perceptions Among Vietnamese Smallholder Tea Farmers and Implications for Environmental Management
Source: Environ Manage. 2026 Jun 26;76(7):228. doi: 10.1007/s00267-026-02534-w (PMC13309507; doi:10.1007/s00267-026-02534-w)
Supplement: Supplementary file 1 — Supplementary information [file 267_2026_2534_MOESM1_ESM.docx]

**Supplementary Information** **for “Nature-Related Risk Perceptions Among Vietnamese Smallholder Tea Farmers and Implications for Environmental Management”**

*Thi-Hoa Vu^1,2^, Brett A. Bryan^1^, Kelly K. Miller^1^, Lai Ming Lam^1^, Carla Archibald ^1^*

1. Deakin University, Melbourne Burwood Campus, 221 Burwood Highway, Burwood, Victoria 3125, Australia.
2. Thai Nguyen University of Agriculture and Forestry, Thai Nguyen, Vietnam

**Table of Contents:**

[Table S1- LEAP-informed mapping of ecosystem interfaces, dependency–impact pathways, and physical natural-related risks in smallholder tea farming 2](#_Toc231213064)

[Table S2- Variables and measurement scales 4](#_Toc231213065)

[Table S3- Selection of explanatory variables across perceived nature-related risk models. 9](#_Toc231213066)

[Table S4- Normality diagnostics for perceived risk variables 12](#_Toc231213067)

[Table S5- Summary of themes on farmers’ perceived drivers of ecosystem service changes and impacts of nature-related risks on tea production. 13](#_Toc231213068)

[Table S6: Results of multiple linear regression models for perceived nature-related risks. 15](#_Toc231213069)

## Table S1- LEAP-informed mapping of ecosystem interfaces, dependency–impact pathways, and physical natural-related risks in smallholder tea farming

This table presents the physical nature-related risks identified through the TNFD LEAP framework. LEAP-informed mapping of ecosystem interfaces, dependency–impact pathways, and physical natural-related risks in smallholder tea farming. Biome categories follow the TNFD biome guidance, and ecosystem service names follow ENCORE terminology as adopted in the TNFD food and agriculture sector guidance (TNFD, 2024). Dependency pathways refer to how tea production relies on nature, while impact pathways refer to how tea production affects nature. The Prepare element was not applied, as it concerns disclosure rather than risk identification.

| **Realm and biome** | **Environmental assets** | **Ecosystem services** | **Dependency pathway** | **Impact pathway** | **Physical nature-related risks** |
| --- | --- | --- | --- | --- | --- |
| ***(Locate)*** | ***(Evaluate)*** | ***(Evaluate)*** | ***(Evaluate)*** | ***(Evaluate)*** | ***(Assess)*** |
| Land realm: Intensive land-use systems | Soil; biodiversity; natural enemy communities | Soil quality regulation; soil and sediment retention; biological control | Tea depends on fertile, stable soils and biological control | Monoculture, slope cultivation, pesticides, and fertilizers degrade soil and biodiversity | Soil degradation; landslides; increased pest and disease occurrence; increased weed occurrence |
| Freshwater realm: rivers, streams, and on-farm water systems | Surface water; groundwater; freshwater ecosystems | Water supply; water purification; water-flow regulation | Tea depends on sufficient, clean water | Water use, runoff, pesticides, and fertilizers reduce water quantity and quality | Water scarcity; water quality decrease |
| Atmospheric systems | Atmospheric systems; climate system | Rainfall pattern regulation; local climate regulation; global climate regulation | Tea depends on stable rainfall, temperature, and seasonal water flows | Fertilizer use may affect atmospheric systems through air pollutants and greenhouse gas emissions | Temperature changes; rainfall pattern changes; increased consecutive hot days; increased consecutive cold days |

##

## Table S2- Variables and measurement scales

This table summarises the key variables included in the analysis, along with their definitions and measurement scales.

| **Main theme** | **Variable names** | **Description/survey questions** | **Scale** | **Reference** |
| --- | --- | --- | --- | --- |
| **Nature-related risk perception** | | | |  |
| ***Perceived risk likelihood*** | RL_Water Scarcity | How do you perceive the likelihood of the following events occurring at your farm in the next 20 years? | 1-5 Likert scale  (No chance of risk to Very high chance of risk) | Dang et al. (2014)  Duong et al. (2019)  Grothmann and Patt (2005) |
|  | RL_Water Quality Decrease |  |  |  |
|  | RL_Soil Degradation |  |  |  |
|  | RL_Landslides |  |  |  |
|  | RL_Increased Weeds Occurrence |  |  |  |
|  | RL_Increased Pest and Disease Occurrence |  |  |  |
|  | RL_Temperature Changes |  |  |  |
|  | RL_Rainfall Pattern Changes |  |  |  |
|  | RL_Increased Consecutive Cold Days |  |  |  |
|  | RL_Increased Consecutive Hot Days |  |  |  |
| ***Perceived risk severity to tea production*** | RS_Water Scarcity | How do you perceive these events' impact on your tea production in the next 20 years if there are no changes in terms of adaptation? | 1-5 Likert scale  (No impact to Very high impact) | Dang et al. (2014)  Duong et al. (2019) |
|  | RS_Water Quality Decrease |  |  |  |
|  | RS_Soil Degradation |  |  |  |
|  | RS_Landslides |  |  |  |
|  | RS_Increased Weeds Occurrence |  |  |  |
|  | RS_Increased Pest and Disease Occurrence |  |  |  |
|  | RS_Temperature Changes |  |  |  |
|  | RS_Rainfall Pattern Changes |  |  |  |
|  | RS_Increased Consecutive Cold Days |  |  |  |
|  | RS_Increased Consecutive Hot Days |  |  |  |
| **Factors influencing farmers' risk perception** | | | |  |
| ***Socio-economic and farm characteristics*** | Farm Size | Total land area used for tea cultivation | ha | Dang et al. (2014)  Duong et al. (2019)  Savari et al. (2025) |
|  | Household Size | Number of individuals living in the household, including the respondent | Number of people |  |
|  | Gender | Gender of the respondent | Female (1); Male (0) |  |
|  | Age | Age of the respondent | Years |  |
|  | Farming Experience | Number of years the respondent has cultivated tea | Yeas |  |
|  | Education Level | The highest level of formal education completed by the respondent | No formal education (1)  Primary school (2); Secondary school (3); High School (4); Tertiary (5) |  |
|  | Annual Income | Total annual income of the household from all sources | VND |  |
|  | Number of Livelihood Activities | All income-generated activities | Number of activities |  |
|  | Number of Group Memberships | Number of social groups the participants are members of. | Number of groups |  |
| ***Nature-related risk experience*** |  |  |  |  |
| *Experienced change in ecosystem services* | Change in Water Availability | How has the environment changed recently compared to when you started your farm? | Decreased (1) No change (2) Increased (3) |  |
|  | Change in Water Quality |  |  |  |
|  | Change in Soil Condition |  |  |  |
|  | Change in Pest and Disease Occurrence |  |  |  |
|  | Change in Weed Occurrence |  |  |  |
|  | Change in Temperature |  |  |  |
|  | Change in Number of Consecutive Hot Days |  |  |  |
|  | Change in Number of Consecutive Cold Days |  |  |  |
| *Experienced nature-related risk impacts* | RE_Water Scarcity | How would you rate the negative impacts of these events on your tea production? | 1-5 Likert scale  (No impact to Very high impact) | Wang et al. (2025)  Shrestha et al. (2022) |
|  | RE_Water Quality Decrease |  |  |  |
|  | RE_Soil Degradation |  |  |  |
|  | RE_Landslides |  |  |  |
|  | RE_Increased Weeds Occurrence |  |  |  |
|  | RE_Increased Pest and Disease Occurrence |  |  |  |
|  | RE_Temperature Changes |  |  |  |
|  | RE_Rainfall Pattern Changes |  |  |  |
|  | RE_Increased Consecutive Cold Days |  |  |  |
|  | RE_Increased Consecutive Hot Days |  |  |  |
| ***Farming practices*** | Chemical Fertilizer Application | Proportion of fertilizer input made up of chemical (synthetic) fertilizers. | % | Wheeler et al. (2021)  Bui and Nguyen (2021)  Bitew and Minale (2025) |
|  | Chemical Pesticide Use | Proportion of pesticide input made up of chemical (synthetic) fertilizers. | % |  |
|  | Organic/Biofertilizer Use | Proportion of fertilizer input made up of organic/bio fertilizer. | % |  |
|  | Organic Pesticide Use | Proportion of total fertilizer input made up of organic/bio fertilizer. |  |  |
|  | Tea Farming Composition Type | The structural composition of the farm based on land use. | All tea crops (4) Primarily tea with some scattered trees (3) Tea with some small habitat patches (2) Tea integrated with natural habitat (1) |  |
|  | Water Storage | Have you used any of the following methods to adapt to changes of environment at your farm? | Yes (1); No (0) |  |
|  | Irrigation System |  |  |  |
|  | Soil Mulching |  |  |  |
|  | Water Use Efficiency |  |  |  |
|  | Organic/bio Fertilizer |  |  |  |
|  | Intercropping |  |  |  |
|  | Organic/Bio Pesticide |  |  |  |
|  | Integrated Pest Management (IPM) |  |  |  |
|  | Planting Schedule Adjustment |  |  |  |
|  | Climate-Resilient Tea Varieties |  |  |  |
| ***Perceived knowledge on nature-related risks*** | Knowledge of Water Scarcity | You are knowledgeable about how weather variabilities will impact tea production. | 1-5 Likert scale  (Strongly disagree to Strongly agree) |  |
|  | Knowledge of Soil Degradation | You are aware of how to assess soil conditions for better management. |  |  |
|  | Knowledge of Weeds, Pests and Disease | You know different incidences of weeds, pests, and diseases impact tea production. |  |  |
|  | Knowledge of Climate Change | You are knowledgeable about how weather variabilities will impact tea production. |  |  |
| ***Government support to adapt to nature-related risks*** | Gov. Support: Water Management | The government provides free technical assistance for enhancing water conservation practices. |  | Dang et al. (2014)  Duong et al. (2019) |
|  | Gov. Support: Soil Management | The government provides free technical assistance for enhancing soil conservation practices. |  |  |
|  | Gov. Support: Integrated Pest Management (IPM) | The government provides guidelines on integrated pest management |  |  |
|  | Gov. Support: Climate-Resilient Cultivars | The government offers free technical advice to find less climate-sensitive cultivars for my land. |  |  |
|  | Gov. Support: Climate Adaptation | The government offers free guidance on adjusting farming practices to cope with changing weather conditions. |  |  |
|  | Gov. Support: Financial Assistance | The government provides subsidies for acquiring facilities to implement adaptation measures. |  |  |
|  | Gov. Support: Organic Subsidy | The government offers subsidies for purchasing organic or bio-fertilizers and pesticides. |  |  |
| ***Information about nature-related risks*** |  |  |  | Dang et al. (2014)  Duong et al. (2019)  Ho et al. (2018) |
| *Type of information received* | Received Information on Water Scarcity | You have been informed about potential water scarcity in your area | 1-5 Likert scale  (Strongly disagree to Strongly agree) |  |
|  | Received Information on Soil Management | You have been informed about the condition of the soil. |  |  |
|  | Received Information on Weeds, Pests, and Diseases | You have information on pests, weeds, and disease incidence at your farm. |  |  |
|  | Received Information on Climate Change | You have access to information about changes in your local climate. |  |  |
| *Sources of information* | Usefulness of Info from Public Media | The usefulness of nature-related risk information from different sources. |  | Dang et al. (2014)  Duong et al. (2019) |
|  | Usefulness of Info from Friends, Relatives & Neighbours |  | 1-5 Likert scale  (Not useful at all to extremely useful) |  |
|  | Usefulness of Info from Local Authorities |  |  |  |
|  | Usefulness of Info from Training Courses |  |  |  |
|  | Usefulness of Info from Social Media |  |  |  |
| ***Belief in nature-related risks*** | Belief in Climate Change | Climate variability is occurring more frequently. | 1-5 Likert scale  (Strongly disagree to Strongly agree) |  |
|  | Belief in Water Scarcity | Your tea farm is adversely impacted by water scarcity. |  |  |
|  | Belief in Soil Degradation | Soil quality is declining, and it is adversely affecting tea output. |  |  |
|  | Belief in Weeds, Pests, and Diseases | Pest and disease management is becoming increasingly complex due to their proliferation. |  |  |

## Table S3- Selection of explanatory variables across perceived nature-related risk models.

Table S2 presents the set of explanatory variables included in the regression models for each perceived nature-related risk. Columns represent individual risk types, while rows list candidate explanatory variables grouped by socio-demographic characteristics, risk experience, observed changes, farming practices, knowledge, institutional support, information access, and beliefs. A value of 1 indicates that the variable was included in the model for the corresponding risk, whereas 0 indicates it was not.

| **Perceived risks**  **Explanatory variables** | **Temperature changes** | **Changes in rainfall patterns** | **Consecutive cold days** | **Consecutive hot days** | **Pest and disease occurrence** | **Weed occurrence** | **Landslides** | **Soil degradation** | **Water scarcity** | **Water quality decrease** | **Overall Nature-related risk** |
| --- | --- | --- | --- | --- | --- | --- | --- | --- | --- | --- | --- |
| Farm Size | 1 | 1 | 1 | 1 | 1 | 1 | 1 | 1 | 1 | 1 | 1 |
| Household Size | 1 | 1 | 1 | 1 | 1 | 1 | 1 | 1 | 1 | 1 | 1 |
| Gender | 1 | 1 | 1 | 1 | 1 | 1 | 1 | 1 | 1 | 1 | 1 |
| Age | 1 | 1 | 1 | 1 | 1 | 1 | 1 | 1 | 1 | 1 | 1 |
| Farming Experience | 1 | 1 | 1 | 1 | 1 | 1 | 1 | 1 | 1 | 1 | 1 |
| Education Level | 1 | 1 | 1 | 1 | 1 | 1 | 1 | 1 | 1 | 1 | 1 |
| Annual Income | 1 | 1 | 1 | 1 | 1 | 1 | 1 | 1 | 1 | 1 | 1 |
| Livelihood Activities | 1 | 1 | 1 | 1 | 1 | 1 | 1 | 1 | 1 | 1 | 1 |
| Number of Group Memberships | 1 | 1 | 1 | 1 | 1 | 1 | 1 | 1 | 1 | 1 | 1 |
| Exp. water scarcity | 1 | 1 | 0 | 1 | 1 | 1 | 0 | 0 | 1 | 0 | 1 |
| Exp. water quality decrease | 0 | 0 | 0 | 0 | 0 | 1 | 0 | 0 | 0 | 1 | 1 |
| Exp. soil degradation | 0 | 1 | 0 | 1 | 1 | 1 | 1 | 1 | 1 | 1 | 1 |
| Exp. landslides | 0 | 0 | 0 | 0 | 0 | 0 | 1 | 1 | 0 | 0 | 1 |
| Exp. weed occurrence | 0 | 0 | 0 | 0 | 0 | 1 | 0 | 0 | 0 | 0 | 1 |
| Exp. pest & disease occurrence | 1 | 1 | 0 | 0 | 1 | 0 | 0 | 0 | 0 | 0 | 1 |
| Exp. temperature changes | 1 | 1 | 1 | 1 | 1 | 1 | 0 | 0 | 1 | 0 | 1 |
| Exp. rainfall changes | 1 | 1 | 1 | 1 | 1 | 1 | 1 | 0 | 1 | 0 | 1 |
| Exp. cold days | 1 | 1 | 1 | 0 | 1 | 0 | 0 | 0 | 0 | 0 | 1 |
| Exp. hot days | 1 | 1 | 0 | 1 | 1 | 1 | 0 | 0 | 1 | 0 | 1 |
| Obs. water availability | 1 | 1 | 1 | 1 | 0 | 0 | 0 | 0 | 1 | 0 | 1 |
| Obs. water quality | 0 | 0 | 0 | 0 | 0 | 0 | 0 | 0 | 0 | 1 | 1 |
| Obs. soil condition | 0 | 0 | 0 | 0 | 1 | 1 | 1 | 1 | 0 | 1 | 1 |
| Obs. pests & diseases | 0 | 0 | 0 | 0 | 1 | 0 | 0 | 0 | 0 | 0 | 1 |
| Obs. weeds | 0 | 0 | 0 | 0 | 0 | 1 | 0 | 0 | 0 | 0 | 1 |
| Obs. temperature | 1 | 1 | 1 | 1 | 1 | 1 | 1 | 0 | 1 | 0 | 1 |
| Obs. hot days | 1 | 1 | 1 | 1 | 1 | 1 | 1 | 0 | 1 | 0 | 1 |
| Obs. cold days | 1 | 0 | 1 | 0 | 1 | 0 | 0 | 0 | 0 | 0 | 1 |
| Chemical Fertilizer Use | 0 | 0 | 0 | 0 | 1 | 1 | 0 | 1 | 0 | 1 | 1 |
| Chemical Pesticide Use | 0 | 0 | 0 | 0 | 1 | 1 | 0 | 1 | 0 | 1 | 1 |
| Biofertilizer Use | 0 | 0 | 0 | 0 | 1 | 1 | 0 | 1 | 0 | 1 | 1 |
| Organic Pesticide Use | 0 | 0 | 0 | 0 | 1 | 0 | 0 | 1 | 0 | 1 | 1 |
| Farm composition | 1 | 1 | 1 | 1 | 0 | 0 | 0 | 1 | 1 | 0 | 1 |
| Water Storage | 1 | 1 | 1 | 1 | 1 | 0 | 0 | 1 | 1 | 1 | 1 |
| Irrigation System | 1 | 1 | 1 | 1 | 1 | 0 | 0 | 1 | 1 | 1 | 1 |
| Soil Mulching | 1 | 1 | 0 | 1 | 0 | 1 | 0 | 1 | 1 | 0 | 1 |
| Water Use Efficiency | 1 | 1 | 1 | 1 | 0 | 0 | 0 | 0 | 1 | 0 | 1 |
| Organic/bio Fertilizer | 0 | 0 | 0 | 0 | 0 | 0 | 0 | 1 | 0 | 0 | 1 |
| Intercropping | 1 | 1 | 0 | 1 | 1 | 0 | 1 | 1 | 1 | 0 | 1 |
| Organic/Bio Pesticide | 0 | 0 | 0 | 0 | 1 | 0 | 0 | 0 | 0 | 0 | 1 |
| Integrated Pest Management | 0 | 0 | 0 | 0 | 1 | 0 | 0 | 0 | 0 | 0 | 1 |
| Planting Schedule Adjustment | 1 | 1 | 1 | 1 | 1 | 0 | 0 | 0 | 0 | 0 | 1 |
| Climate-Resilient Tea Varieties | 1 | 1 | 1 | 1 | 0 | 0 | 0 | 0 | 0 | 0 | 1 |
| Know. water scarcity | 0 | 1 | 0 | 1 | 0 | 0 | 0 | 0 | 1 | 0 | 1 |
| Know. soil degradation | 0 | 0 | 0 | 0 | 0 | 0 | 0 | 1 | 0 | 0 | 1 |
| Know. weeds & pests | 0 | 1 | 0 | 0 | 1 | 1 | 0 | 0 | 0 | 0 | 1 |
| Know. climate change | 1 | 1 | 1 | 1 | 1 | 1 | 1 | 1 | 1 | 0 | 1 |
| Gov. water support | 1 | 1 | 1 | 1 | 0 | 0 | 0 | 0 | 1 | 1 | 1 |
| Gov. soil mgmt. | 0 | 0 | 0 | 0 | 0 | 0 | 0 | 1 | 0 | 0 | 1 |
| Gov. IPM support | 0 | 0 | 0 | 0 | 1 | 0 | 0 | 0 | 0 | 0 | 1 |
| Gov. resilient cultivars | 1 | 1 | 1 | 1 | 0 | 0 | 0 | 0 | 0 | 0 | 1 |
| Gov. climate support | 1 | 1 | 1 | 1 | 0 | 0 | 0 | 0 | 0 | 0 | 1 |
| Gov. financial support | 1 | 1 | 1 | 1 | 1 | 1 | 1 | 1 | 1 | 1 | 1 |
| Gov. organic subsidy | 0 | 0 | 0 | 0 | 0 | 0 | 0 | 1 | 0 | 0 | 1 |
| Info. water scarcity | 0 | 1 | 1 | 1 | 0 | 0 | 0 | 0 | 1 | 0 | 1 |
| Info. soil degradation | 0 | 0 | 0 | 0 | 0 | 0 | 0 | 1 | 0 | 0 | 1 |
| Info. weeds & pests | 0 | 1 | 0 | 0 | 1 | 0 | 0 | 0 | 0 | 0 | 1 |
| Info. climate change | 1 | 1 | 1 | 1 | 1 | 1 | 1 | 0 | 1 | 0 | 1 |
| Info. public media | 0 | 1 | 1 | 1 | 1 | 1 | 1 | 1 | 1 | 1 | 1 |
| Info. friends/neighbours | 1 | 1 | 1 | 1 | 1 | 1 | 1 | 1 | 1 | 1 | 1 |
| Info. authorities | 1 | 1 | 1 | 1 | 1 | 1 | 1 | 1 | 1 | 1 | 1 |
| Info. training | 1 | 1 | 1 | 1 | 1 | 1 | 1 | 1 | 1 | 1 | 1 |
| Info. social media | 1 | 1 | 1 | 1 | 1 | 1 | 1 | 1 | 1 | 1 | 1 |
| Bel. climate change | 1 | 1 | 1 | 1 | 1 | 0 | 0 | 0 | 0 | 0 | 1 |
| Bel. water scarcity | 0 | 1 | 0 | 1 | 0 | 0 | 0 | 0 | 1 | 0 | 1 |
| Bel. soil degradation | 0 | 0 | 0 | 0 | 0 | 0 | 1 | 1 | 1 | 1 | 1 |
| Bel. weeds & pests | 0 | 1 | 1 | 1 | 1 | 1 | 0 | 0 | 0 | 0 | 1 |

## Table S4- Normality diagnostics for perceived risk variables

This table reports the results of normality tests for perceived risk variables, including Shapiro–Wilk statistics, skewness, and kurtosis. Skewness and kurtosis indicated approximately symmetric distributions for most variables. Although Shapiro–Wilk tests were statistically significant, this is attributed to the sensitivity of the test to large sample sizes. Overall, the distribution of variables was approximately symmetric, supporting the use of linear regression models.

| **Perceived risk variable** | **Min** | **Median** | **Mean** | **Max** | **Skewness** | **Kurtosis** | **Shapiro–Wilk p-value** |
| --- | --- | --- | --- | --- | --- | --- | --- |
| Changes in temperature | 2 | 15 | 13.96 | 25 | 0.143 | 2.567 | 3.3e-08 |
| Changes in rainfall patterns | 3 | 12 | 13.65 | 25 | 0.26 | 2.563 | 2.49e-08 |
| Consecutive cold days | 1 | 9 | 10.7 | 25 | 0.467 | 2.405 | 2.27e-09 |
| Consecutive hot days | 4 | 16 | 15.21 | 25 | 0.129 | 2.658 | 2.05e-09 |
| Pest and disease occurrence | 2 | 16 | 14.67 | 25 | -0.085 | 2.656 | 3.44e-09 |
| Weed occurrence | 1 | 9 | 10.71 | 25 | 0.341 | 2.137 | 1.7e-09 |
| Landslides | 1 | 6 | 8.21 | 25 | 1.022 | 3.093 | 1.27e-15 |
| Soil degradation | 1 | 10 | 11.17 | 25 | 0.509 | 2.567 | 4.33e-09 |
| Water scarcity | 1 | 12 | 12.21 | 25 | 0.091 | 2.399 | 1.38e-07 |
| Water quality decline | 1 | 12 | 11.79 | 25 | 0.257 | 2.391 | 2.16e-07 |
| Overall nature-related risk | 3.3 | 12.05 | 12.23 | 22.1 | 0.324 | 3.637 | 0.00268 |

The descriptive statistics indicate that perceived risk scores vary across risk types, with values ranging from 1 to 25, consistent with the construction of the risk index. Most variables show relatively symmetric distributions, with skewness values close to zero (Hatem et al., 2022). However, landslides exhibit a notably higher positive skew (1.022), indicating that most farmers reported low to moderate risk, while a smaller number perceived very high risk. Kurtosis values are generally close to 3, suggesting distributions that approximate normality (Hatem et al., 2022), with the exception of overall risk, which shows slightly higher kurtosis (3.637). Shapiro–Wilk tests are statistically significant for all variables; however, this is expected given the sample size (n = 312) and the sensitivity of the test.

## Table S5- Summary of themes on farmers’ perceived drivers of ecosystem service changes and impacts of nature-related risks on tea production.

This section presents coded qualitative responses from farmer interviews, analysed using a deductive approach to identify perceived drivers of changes in ecosystem services and the impacts of nature-related risks on tea production.

| **Ecosystem changes and nature-related risks** | **Ecosystem change drivers and impacts** | **Comment examples** | **Number of mentions (n)** |
| --- | --- | --- | --- |
| **Farmer-perceived drivers of changes in ecosystem services** | | | |
| Perceived climate variability (temperature, rainfall pattern, consecutive hot days, and cold days) | Climate change | “*I have noticed the climate is changing. Sometimes it gets too hot, and the rain and floods come unpredictably. It is hotter now because of climate change*”- Participant 154. | 70 |
| Increased pest and disease occurrence | Climate-driven increase in pest and disease prevalence | “*Hot weather increases pests such as leaf rollers and red spiders; heavy rain leads to fungal diseases and mosquito bugs*”- Participant 9 | 82 |
|  | Low-quality of fertilizer inputs and pest resistance | “*Unregulated fertilizer quality leads to soil contamination and increased pest outbreaks; unverified pesticides result in pests developing resistance*” - Participant 163. | 3 |
| Decreased water availability | Reduced rainfall and rising temperature |  | 21 |
|  | Increased reliance on drilled wells for irrigation | “*Water availability has decreased as more people use drilled wells to irrigate tea*”- Participant 9. | 15 |
|  | Deforestation |  | 7 |
|  | Lack of electricity for irrigation |  | 5 |
| Decreased water quality | Pollution from chemical fertilizers and pesticides. |  | 10 |
| Soil degradation | Use of chemical inputs and long-term cultivation | “*Prolonged cultivation combined with chemical fertiliser use has led to declining soil quality and degradation*”- Participant 221. | 25 |
|  | Changes in rainfall patterns | “*Heavy rainfall washes away fertile topsoil and fertilizer*”- Participant 66. | 9 |
| Improved soil health | Use of organic/bio fertilisers | “*I think my soil health is better because I apply more manure, which improves soil texture, making it looser and more fertile for tea cultivation*”. | 10 |
| **Impacts of nature-related risks on tea production experienced by farmers** | | | |
| Overall impacts of nature-related risks | Yield and quality decline | “*Extreme heat and heavy rain severely affect tea plants, causing them to wilt or die. These conditions also lead to more pests and diseases, ultimately reducing both the quantity of tea produced and its quality, such as turning the brewed tea reddish instead of the usual green*”. | 77 |
|  | Increased cost/labour | “*Nature-related risks significantly affect tea production by reducing yield stability, lowering quality, increasing labour costs, and making cultivation more challenging*”.  “*Soil degradation, widespread weeds, and frequent pest outbreaks make tea cultivation more difficult and require more frequent fertilizing and pesticide spraying*”. | 22 |
| Impacts of consecutive hot days and high temperature | Tea wilting, leaf scorch, pest outbreaks | “*Hot weather causes tea plants to wither, reducing yield, and leads to increased pest infestations such as leaf rollers and red spiders*”. | 74 |
| Impacts of rainfall pattern changes | Increased pest and disease occurrence: Fungal disease, root rot, and tea blister blight | “*Excessive rainfall affects tea plants by causing fungal diseases, bud rot, or bud wilting, which reduces tea yield*”. | 69 |
|  | Soil erosion |  | 10 |
|  | Waterlogging-induced plant loss | “*Heavy rainfall causes waterlogging, leading to the death of tea plants*”. | 5 |
| Impact of consecutive cold days | Slows tea growth | “*Consecutive cold days prevent bud formation, reducing productivity*”. | 6 |
| Impact of erratic weather | Disrupted harvest/processing, fertilizer inefficiency | “*Frequent changes between sunshine and rain disrupt fertilizing and spraying. This causes fertilizers and pesticides to evaporate or be washed away, leads to topsoil erosion, and results in increased input use, labour, and production costs*”. | 6 |
| Impact of water scarcity | Tea wilting or stunted growth | “*Water scarcity results in poor tea quality and low yield*”. | 7 |
| Impact of soil degradation | Root damage and weak plants |  | 5 |
|  | Poor soil health increases pest/disease susceptibility | “*Increased susceptibility to pests and diseases results from weakened plant resilience due to degraded soil health*”. | 10 |
| Positive impacts of nature-related risks | Higher yield due to climate-driven harvest extension | “*The extension of the harvesting period due to warmer weather and shorter winters has led to increased yields*”. | 9 |
|  | Adequate rain boosts growth. |  | 9 |

## Table S6: Results of multiple linear regression models for perceived nature-related risks.

The table presents coefficient estimates (Coef.), standard errors (SE), t-values, and corresponding p-values for each explanatory variable across different perceived risks. To account for multiple comparisons, p-values were adjusted using the false discovery rate (P (FDR)). Statistical significance is reported based on both raw p-values (Sig. (p < 0.05)) and FDR-adjusted p-values (Sig. (FDR < 0.05)), where “Yes” indicates a statistically significant association and “No” indicates a non-significant association. Positive coefficients indicate higher perceived risk associated with the explanatory variable, while negative coefficients indicate lower perceived risk. The analysis code used to generate these results is available at: <https://github.com/hoa40bmt-debug/Thi_Hoa_Vu_Deakin_University>

| **Perceived risks** | **Explanatory Variable** | **Coef.** | **SE** | **t value** | **p** | **p (FDR)** | **Sig. (p<0.05)** | **Sig. (FDR<0.05)** |
| --- | --- | --- | --- | --- | --- | --- | --- | --- |
| Changes of Rainfall | Intercept | 6.95 | 1.70 | 4.09 | p < 0.001 | NA |  |  |
|  | Info. training | 0.53 | 0.16 | 3.30 | 0.00 | 0.01 | Yes | Yes |
|  | Exp. temperature changes | 0.61 | 0.30 | 2.06 | 0.04 | 0.06 | Yes | No |
|  | Exp. cold days | 0.44 | 0.22 | 2.00 | 0.05 | 0.06 | Yes | No |
|  | Age | 0.04 | 0.02 | 1.92 | 0.06 | 0.06 | No | No |
|  | Gov. resilient cultivars | -1.08 | 0.58 | -1.86 | 0.06 | 0.06 | No | No |
| Changes of Temperature | Intercept | 10.25 | 1.19 | 8.62 | p < 0.001 | NA |  |  |
|  | Exp. cold days | 0.54 | 0.21 | 2.52 | 0.01 | 0.04 | Yes | Yes |
|  | Gov. climate support | 1.37 | 0.58 | 2.37 | 0.02 | 0.04 | Yes | Yes |
|  | Info. authorities | 0.52 | 0.24 | 2.21 | 0.03 | 0.04 | Yes | Yes |
|  | Farm Size | -1.33 | 0.68 | -1.97 | 0.05 | 0.05 | Yes | Yes |
| Consecutive Cold Days | Intercept | 8.33 | 2.01 | 4.14 | p < 0.001 | NA |  |  |
|  | Obs. cold days | 1.27 | 0.38 | 3.31 | 0.00 | 0.01 | Yes | Yes |
|  | Gov. climate support | 2.04 | 0.67 | 3.03 | 0.00 | 0.01 | Yes | Yes |
|  | Info. training | 0.51 | 0.18 | 2.82 | 0.01 | 0.01 | Yes | Yes |
|  | Annual Income | -0.95 | 0.34 | -2.76 | 0.01 | 0.01 | Yes | Yes |
|  | Planting Schedule Adjustment | 1.68 | 0.68 | 2.48 | 0.01 | 0.02 | Yes | Yes |
|  | Exp. cold days | 0.56 | 0.25 | 2.28 | 0.02 | 0.03 | Yes | Yes |
|  | Bel. climate change | -0.67 | 0.36 | -1.87 | 0.06 | 0.06 | No | No |
| Consecutive Hot Days | Intercept | 13.10 | 1.44 | 9.13 | p < 0.001 | NA |  |  |
|  | Annual Income | -0.80 | 0.29 | -2.73 | 0.01 | 0.01 | Yes | Yes |
|  | Info. water scarcity | 0.73 | 0.28 | 2.62 | 0.01 | 0.01 | Yes | Yes |
|  | Livelihood Activities | 0.76 | 0.38 | 2.02 | 0.04 | 0.04 | Yes | Yes |
| Landslides | Intercept | 4.89 | 1.18 | 4.13 | p < 0.001 | NA |  |  |
|  | Info. friends/neighbours | 0.72 | 0.30 | 2.39 | 0.02 | 0.03 | Yes | Yes |
|  | Exp. landslides | 0.44 | 0.20 | 2.16 | 0.03 | 0.03 | Yes | Yes |
| Pest and Disease Occurrence | Intercept | 2.85 | 2.05 | 1.39 | 0.17 | NA |  |  |
|  | Exp. soil degradation | 0.72 | 0.21 | 3.43 | 0.00 | 0.00 | Yes | Yes |
|  | Obs. pests & diseases | 1.31 | 0.40 | 3.28 | 0.00 | 0.00 | Yes | Yes |
|  | Exp. rainfall changes | 0.96 | 0.30 | 3.23 | 0.00 | 0.00 | Yes | Yes |
|  | Info. climate change | 0.66 | 0.26 | 2.52 | 0.01 | 0.02 | Yes | Yes |
|  | Info. training | 0.36 | 0.16 | 2.29 | 0.02 | 0.03 | Yes | Yes |
|  | Obs. soil condition | -0.71 | 0.35 | -2.01 | 0.05 | 0.05 | Yes | No |
|  | Number of Group Memberships | 0.68 | 0.36 | 1.87 | 0.06 | 0.06 | No | No |
| Soil Degradation | Intercept | 15.33 | 2.11 | 7.27 | p < 0.001 | NA |  |  |
|  | Exp. soil degradation | 0.84 | 0.22 | 3.75 | 0.00 | 0.00 | Yes | Yes |
|  | Farming Experience | -1.63 | 0.50 | -3.28 | 0.00 | 0.00 | Yes | Yes |
|  | Chemical Fertilizer Use | 0.03 | 0.01 | 2.47 | 0.01 | 0.02 | Yes | Yes |
|  | Livelihood Activities | -0.85 | 0.42 | -2.03 | 0.04 | 0.04 | Yes | Yes |
| Water Quality Decrease | Intercept | 11.73 | 1.43 | 8.23 | p < 0.001 | NA |  |  |
|  | Exp. soil degradation | 1.06 | 0.23 | 4.62 | p < 0.001 | p < 0.001 | Yes | Yes |
|  | Gov. water support | -2.55 | 0.71 | -3.57 | 0.00 | 0.00 | Yes | Yes |
|  | Annual Income | -0.86 | 0.33 | -2.63 | 0.01 | 0.02 | Yes | Yes |
|  | Obs. soil condition | -0.95 | 0.38 | -2.49 | 0.01 | 0.03 | Yes | Yes |
|  | Exp. water quality decrease | 0.41 | 0.18 | 2.33 | 0.02 | 0.03 | Yes | Yes |
|  | Info. training | 0.36 | 0.17 | 2.13 | 0.03 | 0.05 | Yes | Yes |
|  | Farm Size | -1.46 | 0.73 | -1.99 | 0.05 | 0.05 | Yes | No |
|  | Gov. organic subsidy | 1.43 | 0.75 | 1.91 | 0.06 | 0.06 | No | No |
| Water Scarcity | Intercept | 15.84 | 2.56 | 6.18 | p < 0.001 | NA |  |  |
|  | Exp. soil degradation | 0.65 | 0.23 | 2.80 | 0.01 | 0.02 | Yes | Yes |
|  | Irrigation System | -1.80 | 0.71 | -2.53 | 0.01 | 0.02 | Yes | Yes |
|  | Farming Experience | -1.27 | 0.51 | -2.49 | 0.01 | 0.02 | Yes | Yes |
|  | Exp. hot days | 0.65 | 0.30 | 2.17 | 0.03 | 0.03 | Yes | Yes |
|  | Annual Income | -0.71 | 0.33 | -2.14 | 0.03 | 0.03 | Yes | Yes |
| Weeds Occurrence | Intercept | 4.86 | 2.08 | 2.34 | 0.02 | NA |  |  |
|  | Obs. weeds | 1.54 | 0.45 | 3.44 | 0.00 | 0.00 | Yes | Yes |
|  | Annual Income | -1.00 | 0.35 | -2.84 | 0.00 | 0.01 | Yes | Yes |
|  | Exp. hot days | 0.66 | 0.31 | 2.10 | 0.04 | 0.06 | Yes | No |
|  | Chemical Pesticide Use | 0.03 | 0.01 | 2.00 | 0.05 | 0.06 | Yes | No |
|  | Farm Size | 1.52 | 0.79 | 1.92 | 0.06 | 0.06 | No | No |
|  | Gender | 1.28 | 0.67 | 1.91 | 0.06 | 0.06 | No | No |
| Overall nature-related risks | Intercept | 8.13 | 1.69 | 4.82 | p < 0.001 | NA |  |  |
|  | Exp. soil degradation | 0.40 | 0.12 | 3.38 | 0.00 | 0.01 | Yes | Yes |
|  | Exp. hot days | 0.49 | 0.15 | 3.22 | 0.00 | 0.01 | Yes | Yes |
|  | Obs. pests & diseases | 0.71 | 0.23 | 3.09 | 0.00 | 0.01 | Yes | Yes |
|  | Obs. cold days | 0.56 | 0.19 | 2.98 | 0.00 | 0.01 | Yes | Yes |
|  | Info. friends/neighbours | 0.37 | 0.13 | 2.81 | 0.01 | 0.01 | Yes | Yes |
|  | Annual Income | -0.46 | 0.17 | -2.74 | 0.01 | 0.01 | Yes | Yes |
|  | Obs. water quality | -0.60 | 0.23 | -2.59 | 0.01 | 0.02 | Yes | Yes |
|  | Info. weeds & pests | -0.39 | 0.16 | -2.48 | 0.01 | 0.02 | Yes | Yes |
|  | Info. water scarcity | 0.03 | 0.01 | 2.00 | 0.01406 | 0.017184 | Yes | Yes |
|  | Exp. cold days | 1.52 | 0.79 | 1.92 | 0.040383 | 0.044422 | Yes | Yes |
|  | Farming Experience | 1.28 | 0.67 | 1.91 | 0.065066 | 0.065066 | No | No |

**References:**

Bitew, A. B., & Minale, A. S. (2025). Smallholder farmers' perceptions of climate variability and its risks across agroecological zones in the Ayehu watershed, Upper Blue Nile Basin, Ethiopia. *Environmental and Sustainability Indicators*, *25*, 100546. <https://doi.org/10.1016/j.indic.2024.100546>

Bui, H. T. M., & Nguyen, H. T. T. (2021). Factors influencing farmers’ decision to convert to organic tea cultivation in the mountainous areas of northern Vietnam. *Organic Agriculture*, *11*(1), 51-61. <https://doi.org/10.1007/s13165-020-00322-2>

Dang, H. L., Li, E., Nuberg, I., & Bruwer, J. (2014). Farmers’ Perceived Risks of Climate Change and Influencing Factors: A Study in the Mekong Delta, Vietnam. *Environmental Management*, *54*(2), 331-345. <https://doi.org/10.1007/s00267-014-0299-6>

Duong, T. T., Brewer, T. D., Luck, J., & Zander, K. K. (2019). Understanding biosecurity threat perceptions across Vietnamese smallholder farmers in Australia. *Crop Protection*, *117*, 147-155. <https://doi.org/10.1016/j.cropro.2018.11.022>

Grothmann, T., & Patt, A. (2005). Adaptive capacity and human cognition: The process of individual adaptation to climate change. *Global Environmental Change*, *15*(3), 199-213. <https://doi.org/https://doi.org/10.1016/j.gloenvcha.2005.01.002>

Hatem, G., Zeidan, J., Goossens, M., & Moreira, C. (2022). Normality testing methods and the importance of skewness and kurtosis in statistical analysis. *BAU Journal-Science and Technology*, *3*(2), 7.

Ho, V. B., Nanseki, T., & Chomei, Y. (2018). Farmer’s perceptions on agricultural risks and their determinants: The case of tea production in Thai Nguyen province, Vietnam. *Journal of the Faculty of Agriculture, Kyushu University*, *63*(2), 479-485. <https://doi.org/10.5109/1955671>

Savari, M., Khaleghi, B., & Sheheytavi, A. (2025). How to reduce the risk of climate change for the sustainability of rural farmers? Based on evidence from Iran. *Environmental and Sustainability Indicators*, *25*, 100581. <https://doi.org/10.1016/j.indic.2025.100581>

Shrestha, R., Rakhal, B., Adhikari, T. R., Ghimire, G. R., Talchabhadel, R., Tamang, D., Radhika, K. C., & Sharma, S. (2022). Farmers' Perception of Climate Change and Its Impacts on Agriculture [Article]. *Hydrology*, *9*(12), 15, Article 212. <https://doi.org/10.3390/hydrology9120212>

TNFD. (2024). *Taskforce on Nature-related Financial Disclosures: Additional sector guidance – Food and agriculture*. <https://tnfd.global/publication/additional-sector-guidance-food-and-agriculture/>

Wang, P., Zhang, J., Yuan, S., Wu, X., Liu, J., & Zhang, Y. (2025). Socio-demographic determinants of flood risk perception disparities: A comparative study of subjective and objective risks in Nanjing, China. *Water-Energy Nexus*. <https://doi.org/10.1016/j.wen.2025.06.001>

Wheeler, S. A., Nauges, C., & Zuo, A. (2021). How stable are Australian farmers’ climate change risk perceptions? New evidence of the feedback loop between risk perceptions and behaviour. *Global Environmental Change*, *68*, 102274. <https://doi.org/10.1016/j.gloenvcha.2021.102274>
